# Regulatory microRNAs and phasiRNAs of paclitaxel biosynthesis in *Taxus chinensis*

**DOI:** 10.3389/fpls.2024.1403060

**Published:** 2024-05-08

**Authors:** Ming-Sheng Sun, Yan Jia, Xin-Yi Chen, Ji-Shi Chen, Ying Guo, Fang-Fang Fu, Liang-Jiao Xue

**Affiliations:** State Key Laboratory of Tree Genetics and Breeding, Co-Innovation Center for Sustainable Forestry in Southern China, Key Laboratory of Tree Genetics and Biotechnology of Educational Department of China, Nanjing Forestry University, Nanjing, China

**Keywords:** paclitaxel, *Taxus chinensis*, transcription factors, microRNA, phasiRNA, gene regulatory network

## Abstract

Paclitaxel (trade name Taxol) is a rare diterpenoid with anticancer activity isolated from *Taxus*. At present, paclitaxel is mainly produced by the semi-synthetic method using extract of *Taxus* tissues as raw materials. The studies of regulatory mechanisms in paclitaxel biosynthesis would promote the production of paclitaxel through tissue/cell culture approaches. Here, we systematically identified 990 transcription factors (TFs), 460 microRNAs (miRNAs), and 160 phased small interfering RNAs (phasiRNAs) in *Taxus chinensis* to explore their interactions and potential roles in regulation of paclitaxel synthesis. The expression levels of enzyme genes in cone and root were higher than those in leaf and bark. Nearly all enzyme genes in the paclitaxel synthesis pathway were significantly up-regulated after jasmonate treatment, except for *GGPPS* and *CoA Ligase*. The expression level of enzyme genes located in the latter steps of the synthesis pathway was significantly higher in female barks than in male. Regulatory TFs were inferred through co-expression network analysis, resulting in the identification of TFs from diverse families including MYB and AP2. Genes with ADP binding and copper ion binding functions were overrepresented in targets of miRNA genes. The miRNA targets were mainly enriched with genes in plant hormone signal transduction, mRNA surveillance pathway, cell cycle and DNA replication. Genes in oxidoreductase activity, protein-disulfide reductase activity were enriched in targets of phasiRNAs. Regulatory networks were further constructed including components of enzyme genes, TFs, miRNAs, and phasiRNAs. The hierarchical regulation of paclitaxel production by miRNAs and phasiRNAs indicates a robust regulation at post-transcriptional level. Our study on transcriptional and posttranscriptional regulation of paclitaxel synthesis provides clues for enhancing paclitaxel production using synthetic biology technology.

## 1 Introduction

Paclitaxel is a terpenoid compound isolated from *Taxus*, which can promote the formation and stabilization of microtubules, prevent their depolymerization and inhibit cell division. Widely recognized as a first-line clinical drug, paclitaxel demonstrates curative effects on the treatments of breast cancer, ovarian cancer, and melanoma (De Furia, 1997; Yu et al., 2021). The massive extraction of paclitaxel has posed a serious threat to the growth of *Taxus*, resulting in the disastrous reduction of the natural *Taxus* population. Various approaches, including chemical synthesis, semi-chemical synthesis, plant tissue/cell culture, endophytic fungal synthesis and others, have been explored to develop sustainable methods for paclitaxel production (Fett-Neto et al., 1992; Balogu and Kinston, 1999). Many genes in the paclitaxel biosynthetic pathway were also identified, which can be used to screen bottleneck enzymes to optimize metabolic engineering for paclitaxel production (Howat et al., 2014; Kuang et al., 2019; Perez-Matas et al., 2024). The biosynthesis of paclitaxel involves at least 19 steps, from diterpene precursor geranylgeranyl diphosphate (GGPP) to the final product (Hefner et al., 1998; Jarchow-Choy et al., 2014; Liao et al., 2016). The initial step is the cyclization of GGPP to taxane by the key enzyme TS (taxane synthase) (Ansbacher et al., 2018). Subsequently, the tricyclic taxane backbone undergoes extremely complex modifications mediated by many oxygenases, acyltransferases, and benzoyltransferases, including the 2α-, 5α-, 7β-, 9α-, 10β-, and 13α-hydroxylases, and TAT (taxadienol 5α-*O*-acetyl transferase) and DBAT (10-deacetylbaccatin III 10-*O*-acetyltransferase) (Walker and Croteau, 2000; Walker et al., 2000; Jennewein and Croteau, 2001; Jennewein et al., 2001; Walker et al., 2002; Chau and Croteau, 2004; Kaspera and Croteau, 2006; Long et al., 2008). Recently, two key enzymes have been identified for artificial construction of the baccatin III biosynthetic pathway, including Taxane oxetanase 1 (TOT1) representing a previously unknown enzyme mechanism for oxetane ring formation and T9αH for the taxane oxidation of the C9- position (Jiang et al., 2024).

Transcription factors (TFs) play critical roles in the regulation of plant growth, development, and responses to diverse environmental stresses (Singh et al., 2002; Kahle et al., 2005). In *Taxus*, many TFs have been reported to be involved in the regulations of key genes in the paclitaxel biosynthesis pathway (Kuang et al., 2019; Mutanda et al., 2021). *TcMYC2a* (bHLH member) was considered to play an important role in the jasmonate-responsive expression of *TASY*, *TAT*, *DBTNBT*, *T13αOH*, and *T5αOH* genes (Zhang et al., 2018). Members of the MYB family, known for their roles in various secondary metabolite biosynthesis, also contribute to paclitaxel production (Cao et al., 2020; Yu et al., 2022). In the ERF family, a repressor *TcERF12* and an activator *TcERF15* affected paclitaxel biosynthesis by recognizing the GCC-box on the promoter region of the *TS* gene (Zhang et al., 2015b). *TcWRKY1* significantly enhanced the transcription level of *DBAT* (Li et al., 2013). Engineering of single and/or a combination of TFs would tune the expression of multiple enzyme genes symmetrically for paclitaxel generation.

MicroRNAs (miRNAs) are a class of small non-coding single-stranded RNA molecules of approximately 20-24 nucleotides, which mediate the degradation or inhibition of target genes with diverse functions by sequence complementation (Jones-Rhoades, 2012; Shivaprasad et al., 2012; Deng et al., 2018). In *T. chinensis and T. media*, miRNAs have been reported to significantly correlated with genes in paclitaxel biosynthesis, such as *T5H*, *TAT* and *T10H* (Zhang et al., 2015a; Chen et al., 2020). Phased small interfering RNAs (phasiRNAs) are generated through DCL-catalyzed processing of dsRNA (double-stranded RNA) precursors, they are 21- or 24-nucleotide (nt) in length and start from a precisely defined 5' terminus by trigger miRNAs (Johnson et al., 2009; Dukowic-Schulze et al., 2016). The target genes of phasiRNAs play important roles in various transcriptional regulation processes, such as cell formation, meristem formation, cell cycle, anthocyanin synthesis, response to biotic and abiotic stresses, and so on (Fei et al., 2016; Xia et al., 2019; Liu et al., 2020). The phasiRNAs can also target the transcripts of other phasiRNAs, generating self-enhancing regulatory networks. Unraveling the miRNAs and phasiRNAs regulating paclitaxel biosynthetic genes holds the potential to overcome metabolic bottlenecks.

In this study, we systematically identified enzyme genes in the paclitaxel synthesis pathway and characterized their expression patterns in tissues and treatments. TFs, miRNAs, and phasiRNAs were screened for key regulatory genes in paclitaxel synthesis. The constructed regulatory networks would contribute to our understanding of the regulatory mechanisms in paclitaxel production. The interactions between miRNA/phasiRNA and their targeted protein-coding genes provide clues to promote paclitaxel production through the engineering of genes in posttranscriptional regulations.

## 2 Materials and methods

### 2.1 Plant materials

Fresh young leaves of two female and two male individuals were collected from the natural distribution range of *T. chinensis* (109° 52′19′′N, 30° 60′03′′E) in Taiyanghe, Enshi city, Hubei Province, China. The leaf samples were collected in May, 2021. Published transcriptome and small RNA datasets were also downloaded for analysis including forty-two RNAseq samples (PRJNA730337 and PRJNA251671) and three sRNA samples (PRJNA173133 and PRJNA251671).

### 2.2 RNA-seq library sequencing and data processing

RNA-seq library was constructed using fresh young leaves of *T. chinensis.* Total RNA was extracted using the RNA prep Pure Plant Plus Kit according to the manufacturer’s instructions (LC-BIO TECHNOLOGIES (HANGZHOU) CO., LTD., China). Sequencing was performed using the Illumina NovaSeq platform (Illumina, San Diego, CA, USA) and paired-end raw reads were generated. To obtain high-quality reads, adapters and low-quality reads of the raw data were removed using Trimmomatic (version 0.39) (Bolger et al., 2014). RNA-seq reads were mapped onto the reference genome assembly using STAR (version 2.7.9; parameters: -two pass Mode) (Dobin et al., 2013) and the TPM was calculated to evaluate the expression level of each gene using the RSEM (Li and Dewey, 2011) pipeline after averaging some replicated samples. The numbers of sample replicates in tissue, treatment, and sex experiments in the differential expression analyses are six, one, and three, respectively. Significantly differentially expressed genes were evaluated using edgeR (Robinson et al., 2010) with |logFC| > 1 and FDR < 0.05.

### 2.3 sRNA library sequencing and data processing

Fresh young leaves from the individuals described above were collected for small RNA extraction. sRNA sequencing libraries were prepared using TruSeq Small RNA Sample Prep Kits (USA). Single end reads of 50 bp was obtained using Illumina Hiseq2500 platform (LC-BIO TECHNOLOGIES (HANGZHOU) CO., LTD., China). Reads containing adapters and low-quality reads were trimmed using Cutadapt (version 2.10) (Martin, 2011). The reads were firstly aligned to the Rfam database (version 11.0) (Griffiths-Jones et al., 2005) using Bowtie (version 1.3.0) (Langmead, 2010) to remove non-coding RNAs (rRNA, tRNA, snRNA, scRNA, and snoRNA). The processed sRNA data was submitted to the ShortStack program to identify potential miRNA loci (foldsize = 500; mincov = 2; ranmax = 35) (Axtell, 2013). Loci that meet the N15 criteria or the Y criteria are retained. To identify known miRNAs, we compared candidate mature miRNAs with records in miRBase (version 22.1) (Kozomara et al., 2019) using PatMaN (version 1.2) (Mismatch ≤ 4) (Prüfer et al., 2008). The remaining sequences were then aligned to known miRNA precursor sequences to identify potential miRNA*s. All miRNA loci that meet the Y criteria are considered as novel miRNAs.

To identify potential phasiRNAs loci, processed sRNA data was submitted to the PHASIS (version 3.3) pipeline (Kakrana et al., 2017). Combining the previously identified miRNA sequence with phastrigs (the third module in the PHASIS pipeline), all the miRNA triggers of phasiRNAs were identified. The target genes of miRNAs and phasiRNAs were predicted using the online tool psRNATarget with the following criteria: maximum cutoff of score = 3; penalty for G:U pair = 0.5; penalty for other mismatched = 1; extra penalty weight for mismatched in seed region; HSP size = 19; penalty for opening gap = 2; and penalty for extending gap = 0.5 (Dai et al., 2018). TPTM (transcripts per 10 million reads) was calculated to evaluate the expression level of each miRNA and phasiRNA. GO (Harris et al., 2004) and KEGG (Ogata et al., 1999) enrichment analysis of target genes (FDR ≤ 0.05) was performed through the R package clusterProfiler (version 3.18.1) (Yu et al., 2012). The background gene sets were annotated with Swiss-prot database using Diamond software (E-value ≤ 10^-5^) (version 0.9.19) (Buchfink et al., 2021).

### 2.4 Construction of regulatory networks

Protein coding genes in the paclitaxel synthesis pathway in *T. chinensis* were identified using Diamond Blastp (version 0.9.19) search (E-value ≤ 10^-5^) (Buchfink et al., 2021). In co-expression analysis, the quantitative results of transcriptome were applied for Spearman correlation analysis and weighted gene co-expression network analysis (WGCNA) (Langfelder and Horvath, 2008) was performed to identify co-expressed gene pairs (|ρ| ≥ 0.7). TFs of *T. chinensis* were identified at genome wide using iTAK software (Zheng et al., 2016). The regulatory networks of enzyme genes, TFs, miRNAs, and phasiRNAs were constructed by Cytoscape (version 3.8.2) (Shannon et al., 2003).

## 3 Results

### 3.1 Expression patterns of gene families in paclitaxel synthesis in *T. chinensis*

**Figure 1:**
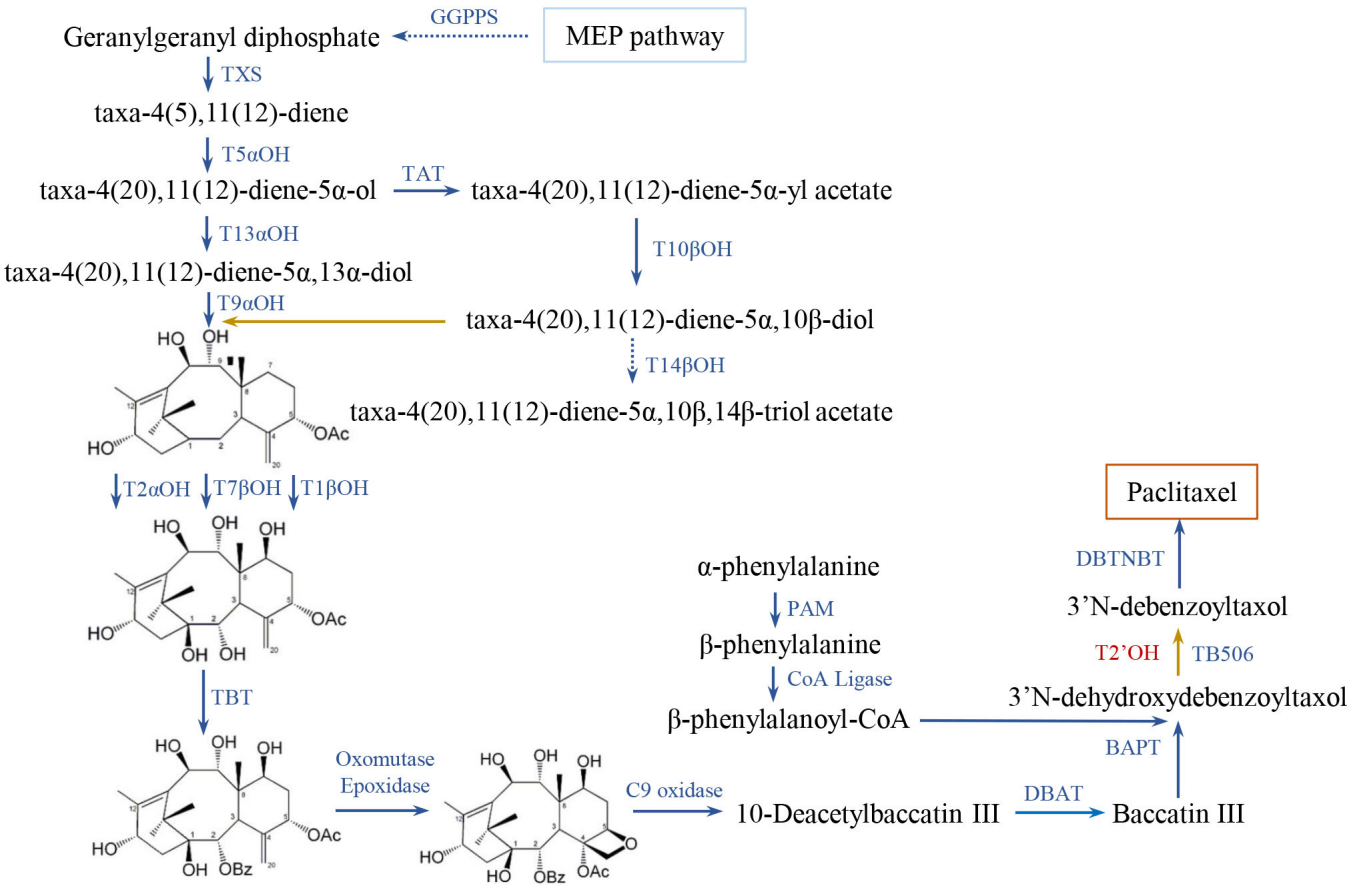
Biosynthetic pathway of paclitaxel in *Taxus*. Identified and unknown enzymes in the pathway are shown in blue and red, respectively. The full names of abbreviations are listed as: GGPPS, geranylgeranyl diphosphate synthase; TXS, Taxadiene synthase; T5αOH, taxane 5α-hydroxylase; TAT, taxadien-5α-ol-O-acetyl-transferase; T13αOH, taxane 13α-hydroxylase; T10βOH, taxane 10β-hydroxylase; T14βOH, taxane 14β-hydroxylase; T2αOH, taxane 2α-hydroxylase; T7βOH, taxane 7β-hydroxylase; TBT, taxane 2α-O-benzoyl transferase; DBAT, 10-deacetyl-baccatin III-10-Oacetyltransferase; PAM, phenylalanine aminomutase; BAPT, 13-O-(3-amino-3-phenylpropanoyl) transferase; DBTNBT, 30-N-debenzoyl-20-deoxytaxol-Nbenzoyltransferase. The metabolic processes were adapted from previous reports (Croteau et al., 2006; Cheng et al., 2021; Zhang et al., 2023; Jiang et al., 2024).

The expression patterns of genes involved in paclitaxel synthesis were investigated to gain insights into their transcriptional regulatory mechanisms (Song et al., 2021). 71 genes of diverse gene families were identified in the whole genome of *T. chinensis* (**Supplementary Tables 1**-**3**). These genes can be categorized into *GGPPS* (geranyl geranyl diphosphate synthase), *T5αOH*, *T13αOH*, *TAT*, *T2αOH*, *T7βOH*, *T10βOH*, *TBT* (taxane-2α-*O*-benzoyl transferase), *BAPT* (C-13-phenylpropanoyl-CoA transferase), *DBTNBT* (3’-*N*-debenzoyl-2’-deoxytaxol *N*-benzoyl transferase), *CoA Ligase*, *PAM* (phenylalanine aminomutase), *T14βOH*, *TB506*, and *TXS* (**Figure 1**). Previously, two gene clusters on chromosome 9 have been reported (Xiong et al., 2021). In analysis, extra *T10βOH_like* genes and *T5αOH_like* genes were identified in the gene cluster located on a 141.69-Mb region of chromosome 9 (616,470,670 - 758,158,182 bp; **Supplementary Table 3**; Xiong et al., 2021).

**Figure 2:**
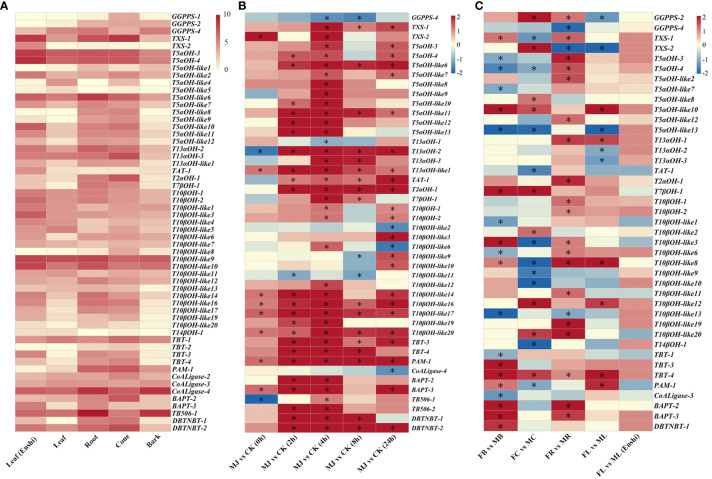
Expression patterns of genes in paclitaxel synthesis in diverse conditions and treatments. **(A)** Expression patterns of differentially expressed genes in leaf, root, cone, and bark tissues of *T. chinensis*. The visualized data has been normalized through log_2_(TPM+1). **(B)** Differential expression patterns of genes in response to methyl jasmonate (MJ) treatment. The samples were from MJ treatment after 0h, 2h, 4h, 8h, and 24h using alcohol as control (CK). **(C)** Differential expression patterns of genes between female and male plants in the tested five samples. FB, female bark; MB, male bark; FC, female cone; MC, male cone; FR, female root; MR, male root; FL, female leaf; ML, male leaf. The differential expression analysis was performed using edgeR with cutoffs |logFC| > 1 and FDR < 0.05. “*” indicates significant up-regulation or significant down-regulation. The order of genes in the heatmap is based on the sequence of chemical reactions in paclitaxel synthesis pathway.

The expression levels of identified genes in *T. chinensis* were further explored in diverse tissues and treatments. The results from tissues indicated most of these genes are expressed at a high level, and the expression of these genes is relatively higher in cone and root in comparison to in leaf and bark (**Figure 2A**). According to previous research results, the application of jasmonate can significantly induce the biosynthesis of paclitaxel and the expression levels of CYP725A subfamily genes in paclitaxel biosynthesis (Xiong et al., 2021). The transcriptional data of *T. chinensis* cell line treated with jasmonate were also included in our analysis. The data were collected at five time points (0h, 2h, 4h, 8h, 24h) with alcohol treatment as the control. The experimental results showed that, except for *GGPPS* and *CoA Ligase*, the expression of almost all enzyme genes in the entire synthesis pathway was significantly up-regulated by jasmonate treatment. The most significant effect was observed at the stage of treatment after four hours (**Figure 2B**). The differential expression patterns of genes in paclitaxel synthesis were also tested between female and male *Taxus* trees. The analysis results showed that the gene expression patterns varied by tissue. In barks, the expression level of enzyme genes located in the latter steps of the synthesis pathway is significantly higher in female plants than in male plants. In cones, the overall expression levels of enzyme genes were lower in male plants, however, the expression patterns were reverse in roots. In leaves, there was only a small portion of genes differentially expressed between female and male plants (**Figure 2C**). Notably, there was no significant expression difference between male leaves and female leaves from Enshi. It is speculated that this may be related to the leaf sampling stage and the physiological state.

### 3.2 Co-expression network of TFs and genes in paclitaxel synthesis pathway

**Figure 3:**
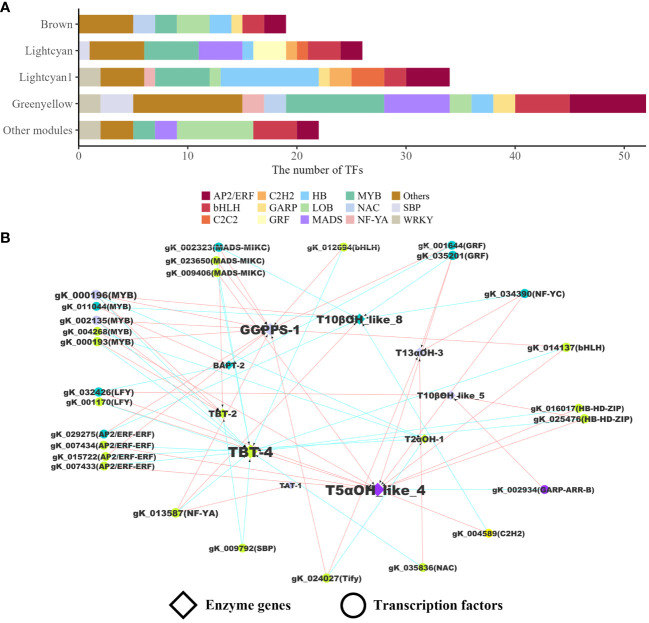
Transcriptional regulatory network in paclitaxel synthesis. **(A)** The number of TFs in WGCNA modules. **(B)** Regulatory network of enzyme genes and TFs. The colors represent WGCNA modules. The pairs of TFs and their targets were linked by arrows. The blue arrows indicate that the weight value between the two genes in WGCNA is greater than 0.3. The red arrows indicate that the absolute value of the Spearman correlation coefficient of two gene expression levels is greater than or equal to 0.7. Each TF in the co-expression network regulates two or more paclitaxel synthesis-related enzyme genes.

To characterize TFs involved in the regulation of paclitaxel synthesis, all members of TF families were identified at the whole genome level based on protein domains. A total of 990 TFs from 60 families were identified. The regulatory pairs between TFs and the target genes were inferred from correlation calculation and WGCNA. We performed Spearman correlation analysis on quantitative results of transcriptome sequencing data to screen and identify the expression associated genes of 71 enzyme genes (|ρ| ≥ 0.7). Most of the TFs were from MYB, AP2/ERF, bHLH, HB, LOB, MADS, and WRKY families (**Figure 3A**). In WGCNA analysis, 29 modules (height > 0.25) were generated based on expression similarity and clustering of gene trees. The enzyme genes in paclitaxel synthesis were mostly present in three modules (Green-yellow, Lightcyan1, and Lightcyan). Finally, 10 enzyme genes and 28 TFs were included in the regulatory networks (**Figure 3B**). These 10 enzyme genes are *GGPPS-1*, *T5αOH_like_4*, *T13αOH-3*, *TAT-1*, *T2αOH-1*, *T10βOH_like_5*, *T10βOH_like_8*, *TBT-2*, *TBT-4* and *BAPT-2*, which are distributed in various steps of paclitaxel biosynthesis. The identified TFs showing co-expressed patterns with enzyme genes can serve as candidate genes to tune the expression of targets at transcription level.

### 3.3 Genome-wide identification of miRNAs in *T. chinensis*

**Figure 4:**
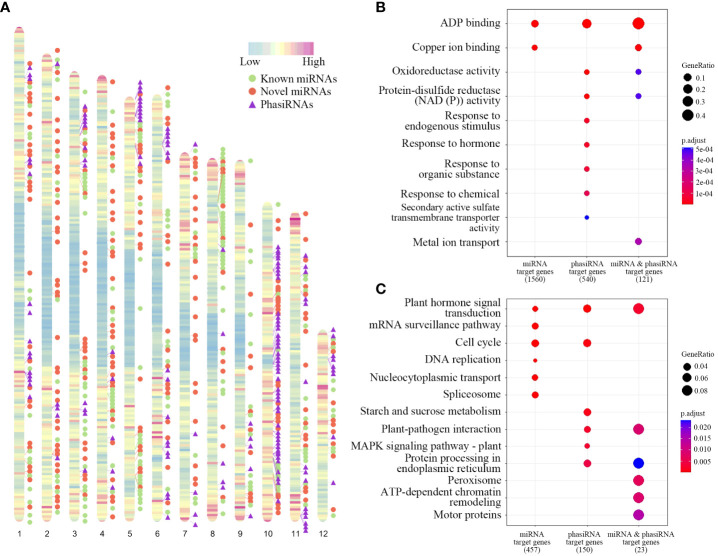
Chromosomal distributions of miRNA and phasiRNA genes and functional enrichment of their targets. **(A)** Heatmap represents the distribution of gene density on chromosomes. The locations of known miRNAs, novel miRNAs, and phasiRNAs were indicated as green dots, red dots, and purple triangles, respectively. **(B, C)** GO **(B)** and KEGG **(C)** enrichment analysis of genes targeted by miRNAs, phasiRNAs, and both of them.

MicroRNAs (miRNAs) genes in *T. chinensis* were identified based on small RNA sequencing and secondary structure prediction. 460 miRNAs from 311 miRNA families were identified (**Supplementary Table 4**), among which 92 and 219 are known and novel families, respectively (**Supplementary Table 5**). The length of miRNAs ranged from 20 nt to 24 nt. The 21-nt miRNAs were most dominant. A significant bias toward U was observed at the first nucleotides of mature miRNA sequences (**Supplementary Table 6**). The distribution patterns of miRNA genes on the chromosomes indicated that they are abundant at arm regions of chromosomes and few of them distribute at the centromere regions (**Figure 4A**).

Target genes were further predicted for all identified miRNAs, among which 49 genes were involved in paclitaxel synthesis (**Supplementary Table 8**). We performed GO enrichment and KEGG enrichment for the predicted target genes (**Figures 4B, C**). GO enrichment results indicated that genes with ADP binding and copper ion binding functions were overrepresented in targets of miRNA genes (**Figure 4C**). KEGG enrichment results show that the target genes of miRNA were mainly enriched with genes in plant hormone signal transduction, mRNA surveillance pathway, cell cycle and DNA replication (**Figure 4C**).

### 3.4 Identification of phasiRNAs in *T. chinensis* genome

PhasiRNAs were also identified at genome level using small RNA reads. A total of 160 21-nt phasiRNAs were obtained, whereas no phasiRNAs with other read sizes were found in the analysis (**Supplementary Table 7**). Among all the phasiRNA genes, 48 are located on chromosome 10, accounting for more than a quarter of the total number. The distribution of phasiRNAs exhibited an enrichment at chromosome regions with high gene density (**Figure 4A**). The target genes of all the phasiRNAs were predicted, among which 18 genes were involved in paclitaxel synthesis (**Supplementary Table 9**). GO analysis results show that genes in ADP binding, oxidoreductase activity, protein-disulfide reductase activity were enriched in targets of phasiRNAs. KEGG enrichment results show that target genes of phasiRNA were mainly enriched in pathways including plant hormone signal transduction, cell cycle, starch and sucrose metabolism, and plant-pathogen interaction (**Figure 4C**). We also performed functional enrichment analysis for the intersection of target genes of miRNAs and phasiRNAs. The miRNA triggers of phasiRNAs were further predicted, resulting the identification of 280 miRNAs targeting all 160 phasiRNAs.

### 3.5 Regulatory network of paclitaxel production mediated by miRNAs and phasiRNAs

**Figure 5:**
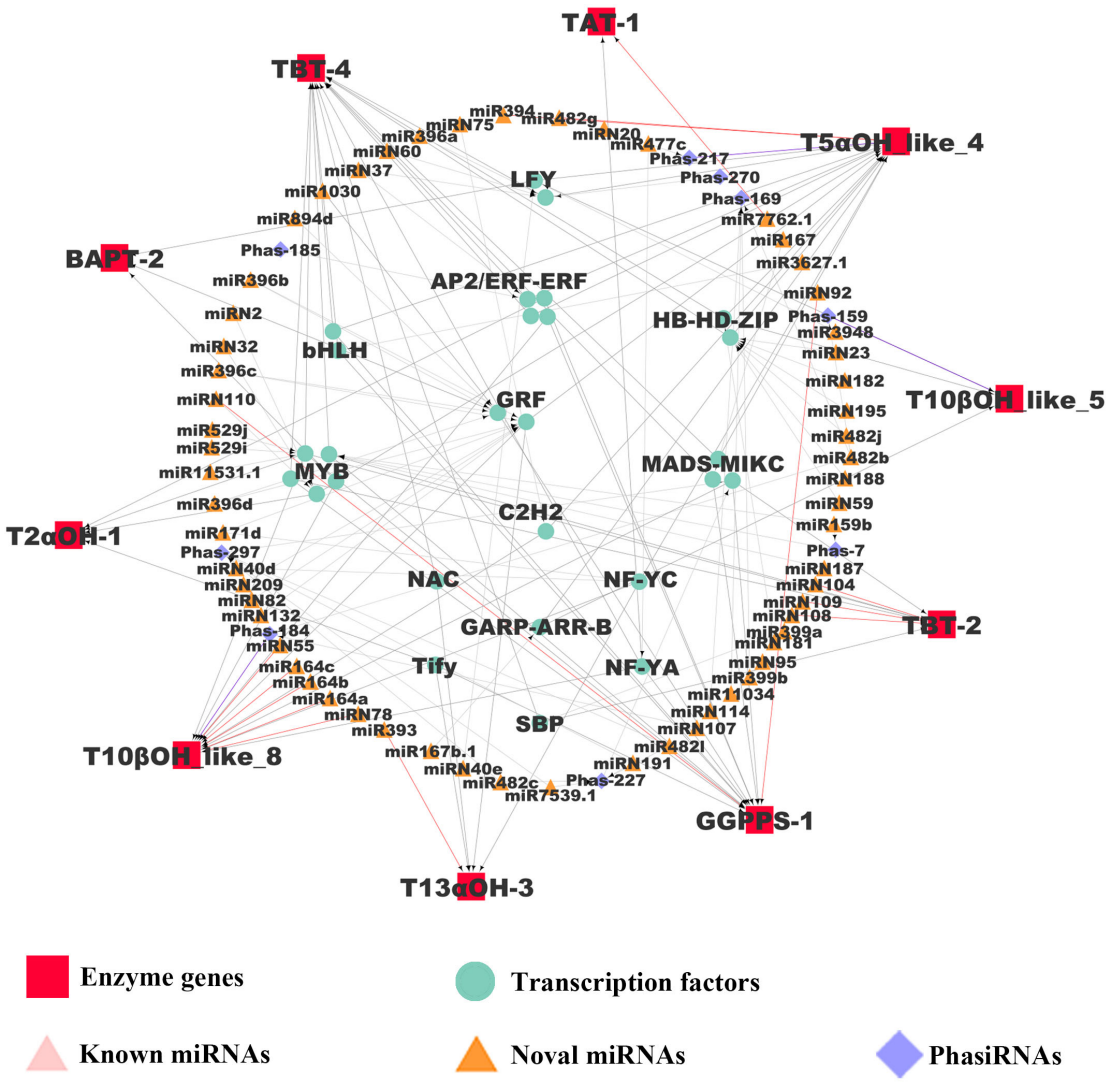
Regulatory network mediated by miRNAs and phasiRNAs in paclitaxel synthesis. The pairs of regulatory genes (miRNAs, phasiRNAs, and TFs) and their target genes were visualized with arrows. Red and purple lines represent miRNA/enzyme gene pairs and phasiRNA/enzyme gene pairs relationships, respectively. Gray lines represent miRNA/TF, phasiRNA/TF and TF/enzyme gene pairs.

The regulatory connections among miRNAs, phasiRAs, TFs, and enzyme genes in paclitaxel synthesis were combined to construct a regulatory network, which exhibits hierarchical structures in gene regulation (**Figure 5**). In the network, 60 miRNAs, 9 phasiRNAs and 14 TFs were inferred to regulated 10 key enzyme genes. Among the miRNAs in the network, 15 miRNAs are present in *T. chinensis* leaf with high abundance (**Table 1**). Further analysis indicated that six miRNAs regulated enzyme genes directly, and the other six miRNA genes regulated the enzyme genes through targeting TFs. Three miRNAs were predicted to trigger the production of phasiRNAs targeting TFs, which in turn regulate the transcriptions of enzymes genes. The hierarchical regulation of paclitaxel production by miRNAs and phasiRNAs indicates a robust regulation at post-transcriptional level.

**Table 1:** Target genes of miRNAs and phasiRNAs with high abundance.

Name	TPTM	Target genes	Note
A. miRNAs targeting enzyme genes			
miRN108	1649830	*TBT-2*	
miR164a	858620	*T10βOH_like_8*	
miRN109	667260	*TBT-2*	
miR482g	552750	*T5αOH_like_4*	
miR164c	518290	*T10βOH_like_8*	
miR7762.1	43890	*TAT-1*	
Phas-217	275530	*T5αOH_like_4*	
B. miRNAs targeting TFs			
miR482j	2172780	*gK_016017* (HB-HD-ZIP)	*TBT-4*, *T5αOH_like_4*
miR396d	378950	*gK_001644* (GRF)	*BAPT-2*, *GGPPS-1*
		*gK_035201* (GRF)	*T10βOH_like_8*, *T13αOH-3*, *T5αOH_like_4*
miR159b	224500	*gK_002135* (MYB)	*TBT-4*, *GGPPS-1*, *TBT-2*
miRN187	112100	*gK_002135* (MYB)	*TBT-4*, *GGPPS-1*, *TBT-2*
miR396b	102640	*gK_001644* (GRF)	*BAPT-2*, *GGPPS-1*
		*gK_035201* (GRF)	*T10βOH_like_8*, *T13αOH-3*, *T5αOH_like_4*
miRN209	44470	*gK_035201* (GRF)	*T10βOH_like_8*, *T13αOH-3*, *T5αOH_like_4*
C. miRNAs targeting phasiRNAs			
miR482c	3957410	Phas-227	*gK_002323* (MADS-MIKC) - *T10βOH_like_8*, *GGPPS-1*, *TBT-2*
		Phas-297	*gK_002323* (MADS-MIKC) - *T10βOH_like_8*, *GGPPS-1*, *TBT-2*
miR482l	934790	Phas-227	*gK_002323* (MADS-MIKC) - *T10βOH_like_8*, *GGPPS-1*, *TBT-2*
		Phas-297	*gK_002323* (MADS-MIKC) - *T10βOH_like_8*, *GGPPS-1*, *TBT-2*
miR159b	224500	Phas-7	*gK_002135*(MYB) - *TBT-4*, *GGPPS-1*, *TBT-2*

For miRNAs targeting TFs, the downstream enzyme genes are listed. For miRNAs targeting phasiRNAs, the target TFs and their downstream enzyme genes are both listed.

## 4 Discussion

### 4.1 Diverse enzyme genes in paclitaxel synthesis in *T. chinensis*

As an anticancer drug, the molecular structure of paclitaxel is complex, with 11 stereocenters and a 17-carbon tetracyclic skeleton structure (Jennewein and Croteau, 2001). Despite many attempts to study its chemical synthesis, the intricate route, challenging reaction conditions, and low synthesis rate have posed significant hurdles for researchers. Consequently, attentions in the community have shifted toward the semi-synthesis method. The intermediate products like 10-deacetyl baccatin III (10-DAB) and baccatin III from taxanes were first extracted and applied for paclitaxel chemical synthesis. The semi-synthesis method is a high-purity, cost-effective approach, which has become the primary method for industrial production (Li et al., 2015). While the synthesis technology has been well studied, the production of paclitaxel remains constrained by limited *Taxus* resources. The large-scale culture of *Taxus* cells and the fermentation of endophytic fungi to produce paclitaxel have been avenues for breakthroughs. In the realm of metabolic engineering, especially with advancements in synthetic biology, successful large-scale synthesis of key natural products like artemisinin and ginsenosides from heterologous sources has been achieved (Ro et al., 2006; Yan et al., 2014; Sanchez-Muñoz et al., 2020; Schneider et al., 2020; Su et al., 2022).

The limiting factor affecting efficiency of paclitaxel semi-synthetic is the full picture of genes in the synthesis pathway. Six previously characterized *Taxus* genes can coordinatively produce key paclitaxel intermediates and serves as a crucial platform for the discovery of the remaining biosynthetic genes (Liu et al., 2024). The screening strategy for the biosynthesis pathway of paclitaxel is constantly being updated, new enzyme genes like *TOT1* and *T9αH1* were innovatively proposed and precisely located in the synthesis pathway (Jiang et al., 2024). In our study, most of the known genes involved in paclitaxel biosynthesis are located on an 80.46-Mb region and a 141.69-Mb region on chromosome 9, which were consistent with previous studies, and the number of identified enzyme genes in the clusters also increased (**Supplementary Table 3**; Xiong et al., 2021). The extra *T10βOH_like* genes and *T5αOH_like* genes identified in the 141.69-Mb region may have unknown novel functions, which requires further exploration and verification to provide insights and candidate genes for paclitaxel semi-synthesis.

### 4.2 Regulatory TFs of paclitaxel synthesis in *T. chinensis*

The biosynthesis pathway of paclitaxel is fine-tuned at transcription level (Li et al., 2020; Li et al., 2022). More and more TFs are recognized as pivotal regulators in the paclitaxel pathway (Kuang et al., 2019; Mutanda et al., 2021). Previous reports have shown that some transcription factor, such as ERF, can bind the promoter of multiple genes in the pathway (Zhang et al., 2015b). At the same time, a single enzyme gene can be regulated by several TFs (Zhang et al., 2015b; Cao et al., 2020). Depending on different metabolic regulation purposes in paclitaxel biosynthesis, different transcription factors are used to construct various transcriptional regulation tools. Researchers are actively exploring diverse TFs through gene function validation to modulate each enzyme gene in the paclitaxel synthesis pathway, paving the way for enhanced transcriptional expression and improved synthesis efficiency. The TFs targeting to genes in paclitaxel pathway can also involve the regulation genes in the growth and development of *T. chinensis* (Li et al., 2022).

The complexity of metabolic pathways often leads to unexpected phenotypes in metabolic engineering. The overexpression of enzyme genes may result in the accumulation of toxic intermediate metabolites, while downregulation and knockout of key genes may lead to the shortage of metabolites required for cell growth (Wu et al., 2020). In addition, the modifying of a single gene may disrupt cell homeostasis, causing destructive effects on cell stress, growth, and division, leading to the decrease in synthesis efficiency of final products (Xu et al., 2020). Simultaneously modification of multiple genes could be ideal for perturbation of metabolic pathways. The metabolic regulatory system composed of TFs has been widely applied in metabolic engineering and synthetic biology due to its ability to globally and dynamically regulate target pathways (Deng et al., 2022). Based on the TFs regulatory network in our study, single and/or a combination of TFs can be knock-outed through genome editing or overexpressed by transgenic approaches to optimize transcription of enzyme genes.

### 4.3 MiRNAs and phasiRNAs regulation of genes related to paclitaxel synthesis in *T. chinensis*

MiRNAs have been reported to play significant roles in plant secondary metabolism (Zhai et al., 2011; Owusu Adjei et al., 2021). In our analysis, many enzyme genes in paclitaxel synthesis are predicted as targets of miRNAs and phasiRNAs, indicating the roles of posttranscriptional regulation in paclitaxel synthesis. Notably, key genes like *GGPPS-1*, *BAPT-2*, *T13αOH-3*, *TBT-4*, *T10βOH_like_5*, and *T2αOH-1* are directly targeted by 15 miRNAs with high abundance in *T. chinensis* leaf. These miRNAs also target to TFs with functions in regulation of plant growth and development. The miRNAs could function as intermediate signals controlling the balance of paclitaxel synthesis and plant development (Ha and Kim, 2014).

The identification of miRNAs and phasiRNAs in paclitaxel synthesis could also provide novel strategies to elevate enzyme genes expression, thereby boosting paclitaxel production. The miRNA sponges and competing endogenous RNAs (ceRNAs) can bind to miRNAs and phasiRNAs to inhibit their functions (Panda, 2018; Smillie et al., 2018). Specific miRNA sponges and ceRNAs can be designed and overexpressed in tissues and cell lines of taxus to block key miRNAs and phaisRNAs, which could release the expression of enzyme genes. Genome editing techniques, such as CRISPR/Cas9 could also be applied to know out the expression of miRNAs and phasiRNAs to improve paclitaxel production (Deng et al., 2022).

## 5 Conclusion

In summary, our comprehensive investigation systematically identified enzyme-encoding genes involved in paclitaxel biosynthesis in *T. chinensis* and their transcriptional expression at diverse tissues. TFs, miRNAs and phasiRNAs were identified, followed by the construction of regulatory networks encompassing enzyme genes, and their upstream regulators. The hierarchical regulation of paclitaxel production by miRNAs and phasiRNAs indicates a robust regulation at post-transcriptional level. This study contributes valuable insights into the regulatory expression patterns of paclitaxel synthesis-related enzyme genes and provide guidance to elevate paclitaxel production.

## Data availability statement

Raw data of RNAseq and sRNAseq of studied samples from Enshi have been deposited in the NCBI SRA under the accession PRJNA1031429.

## Author contributions

M-SS: Data curation, Formal analysis, Investigation, Visualization, Writing – original draft, Writing – review & editing. YJ: Formal analysis, Investigation, Writing – original draft. X-YC: Investigation, Writing – original draft. J-SC: Investigation, Writing – original draft. YG: Investigation, Writing – original draft. F-FF: Methodology, Supervision, Writing – original draft, Writing – review & editing. L-JX: Methodology, Supervision, Writing – original draft, Writing – review & editing.
