# Supplementary material for: Regulatory microRNAs and phasiRNAs of paclitaxel biosynthesis in Taxus chinensis
Source: Front Plant Sci. 2024 May 8;15:1403060. doi: 10.3389/fpls.2024.1403060 (PMC11109412; doi:10.3389/fpls.2024.1403060)
Supplement: Supplementary file 1 [file DataSheet_1.docx]

# Table S1 Known genes encoding enzymes in paclitaxel biosynthesis pathway in *Taxus chinensis*.

| ***T. chinensis* ID** | ***T. yunnanensis* ID** | **Accession number** | **Gene name** | **Description** | **chromosome** | **Start** | **End** | **Strand** |
| --- | --- | --- | --- | --- | --- | --- | --- | --- |
| KI387_000913 | Tyu0088740.1 | AGT51232.1 | BAPT-1 | 13-*O*-(3-amino-3-phenylpropanoyl) transferase | chr1 | 193268852 | 193269640 | - |
| KI387_005354 | Tyu0157660.1 | AEJ84001.1 | BAPT-2 | 13-*O*-(3-amino-3-phenylpropanoyl) transferase | chr2 | 240694345 | 240694986 | + |
| KI387_003225 | Tyu0157670.1 | AEJ84001.1 | BAPT-3 | 13-*O*-(3-amino-3-phenylpropanoyl) transferase | chr1 | 842005649 | 842305283 | + |
| KI387_025352 | Tyu0191170.1 | AGT51232.2 | BAPT-4 | 13-*O*-(3-amino-3-phenylpropanoyl) transferase | chr7 | 783921512 | 783922252 | + |
| KI387_035235 | Tyu0038920.1 | AKA59704.1 | CoA Ligase-1 | coaligase | chr11 | 17056423 | 17070643 | - |
| KI387_028438 | Tyu0114740.1 | AKA59704.1 | CoA Ligase-2 | coaligase | chr9 | 1763797 | 1775627 | - |
| KI387_028426 | Tyu0269590.1 | AKA59704.1 | CoA Ligase-3 | coaligase | chr9 | 668461 | 701137 | + |
| KI387_028882 | Tyu0270180.1 | AKA59704.1 | CoA Ligase-4 | coaligase | chr9 | 81124423 | 81127770 | + |
| KI387_025350 | Tyu0174990.1 | AAM75818.1 | DBTNBT-1 | 3'-*N*-debenzoyl-2'-deoxytaxol-*N*-benzoyltransferase | chr7 | 783866134 | 783867459 | - |
| KI387_034705 | Tyu0259610.1 | AAM75818.1 | DBTNBT-2 | 3'-*N*-debenzoyl-2'-deoxytaxol-*N*-benzoyltransferase | chr10 | 624732378 | 624734159 | + |
| KI387_029317 | Tyu0190990.1 | AAA32797.1 | GGPPS-1 | Geranylgeranyl diphosphate synthase | chr9 | 185645349 | 185646881 | + |
| KI387_027794 | Tyu0148360.1 | AAA32797.1 | GGPPS-2 | Geranylgeranyl diphosphate synthase | chr8 | 666088130 | 666089662 | + |
| KI387_010989 | Tyu0224260.1 | AAA32797.1 | GGPPS-4 | Geranylgeranyl diphosphate synthase | chr3 | 857421284 | 857422241 | + |
| KI387_022619 | Tyu0234180.1 | AAT47186.1 | PAM-1 | Phenylalanine aminomutase | chr7 | 81338883 | 81348526 | - |
| KI387_028600 | Tyu0015680.1 | AAK00946.1 | T10βOH-1 | Taxane 10-beta-hydroxylase | chr9 | 26609189 | 26610694 | - |
| KI387_028601 | Tyu0015690.1 | AAK00946.1 | T10βOH-2 | Taxane 10-beta-hydroxylase | chr9 | 26618048 | 26620050 | - |
| KI387_015265 | Tyu0015390.1 | AAL23619.1 | T13αOH-1 | Taxane 13-alpha-hydroxylase | chr5 | 29920805 | 29927057 | - |
| KI387_028567 | Tyu0015420.1 | AAL23619.2 | T13αOH-2 | Taxane 13-alpha-hydroxylase | chr9 | 23049117 | 23051056 | + |
| KI387_028549 | Tyu0169960.1 | AAL23619.3 | T13αOH-3 | Taxane 13-alpha-hydroxylase | chr9 | 19994272 | 19996422 | + |
| KI387_031460 | Tyu0086220.1 | AAO66199.1 | T14βOH-1 | Taxane 14-beta-hydroxylase | chr9 | 757919766 | 757921392 | - |
| KI387_028599 | Tyu0015660.1 | AAS89065.2 | T2αOH-1 | Taxane 2-alpha-hydroxylase | chr9 | 26464535 | 26466384 | + |
| KI387_028755 | Tyu0130930.1 | AAQ56240.2 | T5αOH-1 | Taxane 5-alpha-hydroxylase | chr9 | 49866964 | 49867451 | - |
| KI387_028803 | Tyu0234990.1 | AAQ56240.2 | T5αOH-3 | Taxane 5-alpha-hydroxylase | chr9 | 55503341 | 55505492 | + |
| KI387_028800 | Tyu0342120.1 | AAQ56240.2 | T5αOH-4 | Taxane 5-alpha-hydroxylase | chr9 | 55349630 | 55350537 | - |
| KI387_028597 | Tyu0015620.1 | AAQ75553.1 | T7βOH-1 | Taxane 7-beta-hydroxylase | chr9 | 26122400 | 26286615 | - |
| KI387_030974 | Tyu0245600.1 | AAF34254.1 | TAT-1 | Taxadien-5α-ol-O-acetyl transferase | chr9 | 676508395 | 676511053 | - |
| KI387_010353 | Tyu0176400.1 | AKH04263.1 | TB506-1 | Trans-cinnamate 4-hydroxylase | chr3 | 720021327 | 720023309 | + |
| KI387_011716 | Tyu0176410.1 | AKH04263.1 | TB506-2 | Trans-cinnamate 4-hydroxylase | chr4 | 27094937 | 27096354 | - |
| KI387_001001 | Tyu0033130.1 | AAG38049.1 | TBT-1 | Taxane 2α-*O*-benzoyl transferase | chr1 | 212346584 | 212348286 | - |
| KI387_000888 | Tyu0078490.1 | AAG38049.1 | TBT-2 | Taxane 2α-*O*-benzoyl transferase | chr1 | 187725690 | 187727457 | - |
| KI387_000915 | Tyu0088730.1 | AAG38049.1 | TBT-3 | Taxane 2α-*O*-benzoyl transferase | chr1 | 193569065 | 193571440 | + |
| KI387_000939 | Tyu0225190.1 | AAG38049.1 | TBT-4 | Taxane 2α-*O*-benzoyl transferase | chr1 | 196812482 | 196814443 | - |
| KI387_028840 | Tyu0060310.1 | AAC49310.1 | TXS-1 | Taxadiene Synthase | chr9 | 72105319 | 72109598 | - |
| KI387_028802 | Tyu0235010.1 | AAC49310.1 | TXS-2 | Taxadiene Synthase | chr9 | 55467291 | 55471405 | - |

# Table S2 CYP725 genes in *Taxus chinensis*.

| ***T. chinensis* ID** | ***T. yunnanensis* ID** | **Uniprot** | **Subject** | | **chromosome** | **Start** | **End** | **Strand** |
| --- | --- | --- | --- | --- | --- | --- | --- | --- |
| KI387_015265 | Tyu0015390.1 | 13-alpha-hydroxylase | | T13αOH_AAL23619.1.fasta\|Q8W4T9\|T13H_TAXCU | chr5 | 29920805 | 29927057 | - |
| KI387_028567 | Tyu0015420.1 | 13-alpha-hydroxylase | | T13αOH_AAL23619.1.fasta\|Q8W4T9\|T13H_TAXCU | chr9 | 23049117 | 23051056 | + |
| KI387_024865 | Tyu0015550.1 | 5-alpha-taxadienol-10-beta-hydroxylase | | T10βOH-CYP725A1_AAK00946.1.fasta\|Q9AXM6\|T10H_TAXCU | chr7 | 685410689 | 685412440 | + |
| KI387_001515 | Tyu0015610.1 | 5-alpha-taxadienol-10-beta-hydroxylase | | T10βOH-CYP725A1_AAK00946.1.fasta\|Q9AXM6\|T10H_TAXCU | chr1 | 313662980 | 313666709 | - |
| KI387_028597 | Tyu0015620.1 | Taxoid 7-beta-hydroxylase | | T7βOH_AAQ75553.1.fasta\|Q6JTJ0\|T7H_TAXCU | chr9 | 26122400 | 26286615 | - |
| KI387_028598 | Tyu0015650.1 | CYP725A9-Taxane 10-beta-hydroxylase | | T5αOH_AAQ56240.2.fasta\|Q6WG30\|T5H_TAXCU | chr9 | 26455040 | 26462359 | - |
| KI387_028599 | Tyu0015660.1 | Taxoid 7-beta-hydroxylase+Taxoid 2-alpha-hydroxylase | | T2αOH_AAS89065.2.fasta\|Q6JD68\|Q6JD68_TAXCA | chr9 | 26464535 | 26466384 | + |
| KI387_028600 | Tyu0015680.1 | 5-alpha-taxadienol-10-beta-hydroxylase | | T10βOH-CYP725A1_AAK00946.1.fasta\|Q9AXM6\|T10H_TAXCU | chr9 | 26609189 | 26610694 | - |
| KI387_028601 | Tyu0015690.1 | 5-alpha-taxadienol-10-beta-hydroxylase | | T10βOH-CYP725A1_AAK00946.1.fasta\|Q9AXM6\|T10H_TAXCU | chr9 | 26618048 | 26620050 | - |
| KI387_024864 | Tyu0024830.1 | 5-alpha-taxadienol-10-beta-hydroxylase | | T10βOH-CYP725A1_AAK00946.1.fasta\|Q9AXM6\|T10H_TAXCU | chr7 | 685396781 | 685399209 | + |
| KI387_030703 | Tyu0024960.1 | `CYP725A12 | | T10βOH-CYP725A1_AAK00946.1.fasta\|Q9AXM6\|T10H_TAXCU | chr9 | 620103736 | 620104758 | + |
| KI387_026089 | Tyu0024990.1 | CYP725A14 | | T10βOH-CYP725A1_AAK00946.1.fasta\|Q9AXM6\|T10H_TAXCU | chr8 | 142833504 | 142835450 | - |
| KI387_030698 | Tyu0025000.1 | CYP725A12 | | T5αOH_AAQ56240.2.fasta\|Q6WG30\|T5H_TAXCU | chr9 | 619756079 | 619756982 | + |
| KI387_030697 | Tyu0025010.1 | CYP725A12 | | T10βOH-CYP725A1_AAK00946.1.fasta\|Q9AXM6\|T10H_TAXCU | chr9 | 619528673 | 619529568 | - |
| KI387_001129 | Tyu0025020.1 | CYP725A12 | | T10βOH-CYP725A1_AAK00946.1.fasta\|Q9AXM6\|T10H_TAXCU | chr1 | 239941118 | 239947688 | - |
| KI387_030692 | Tyu0025050.1 | CYP725A14-10-beta-hydroxylase | | T10βOH-CYP725A1_AAK00946.1.fasta\|Q9AXM6\|T10H_TAXCU | chr9 | 619079075 | 619080176 | - |
| KI387_024704 | Tyu0025060.1 | CYP725A12 | | T10βOH-CYP725A1_AAK00946.1.fasta\|Q9AXM6\|T10H_TAXCU | chr7 | 638166764 | 638170642 | - |
| KI387_030687 | Tyu0025080.1 | Taxane 10-beta-hydroxylase | | T10βOH-CYP725A1_AAK00946.1.fasta\|Q9AXM6\|T10H_TAXCU | chr9 | 618632411 | 618634323 | - |
| KI387_030686 | Tyu0025100.1 | CYP725A12 | | T10βOH-CYP725A1_AAK00946.1.fasta\|Q9AXM6\|T10H_TAXCU | chr9 | 618545892 | 618547205 | - |
| KI387_030673 | Tyu0025120.1 | CYP725A12 | | T10βOH-CYP725A1_AAK00946.1.fasta\|Q9AXM6\|T10H_TAXCU | chr9 | 616470670 | 616471157 | - |
| KI387_031461 | Tyu0025130.1 | CYP725A12 | | T10βOH-CYP725A1_AAK00946.1.fasta\|Q9AXM6\|T10H_TAXCU | chr9 | 758157695 | 758158182 | + |
| KI387_030679 | Tyu0025150.1 | T10H_TAXCU`Taxane 10-beta-hydroxylase | | T5αOH_AAQ56240.2.fasta\|Q6WG30\|T5H_TAXCU | chr9 | 618189129 | 618190194 | + |
| KI387_012225 | Tyu0025160.1 | Taxane 10-beta-hydroxylase | | T10βOH-CYP725A1_AAK00946.1.fasta\|Q9AXM6\|T10H_TAXCU | chr4 | 143321730 | 143323009 | - |
| KI387_023139 | Tyu0042090.1 | Taxoid 7-beta-hydroxylase | | T7βOH_AAQ75553.1.fasta\|Q6JTJ0\|T7H_TAXCU | chr7 | 174326447 | 174326934 | - |
| KI387_023119 | Tyu0050210.1 | Taxane 10-beta-hydroxylase | | T10βOH-CYP725A1_AAK00946.1.fasta\|Q9AXM6\|T10H_TAXCU | chr7 | 172117778 | 172120284 | - |
| KI387_023122 | Tyu0050220.1 | CYP725A11 | | T5αOH_AAQ56240.2.fasta\|Q6WG30\|T5H_TAXCU | chr7 | 172488994 | 172489726 | - |
| KI387_028495 | Tyu0058490.1 | 5-alpha-taxadienol-10-beta-hydroxylase | | T10βOH-CYP725A1_AAK00946.1.fasta\|Q9AXM6\|T10H_TAXCU | chr9 | 11431718 | 11433471 | + |
| KI387_028858 | Tyu0060190.1 | CYP725A19+Taxoid 2-alpha-hydroxylase+T5H | | T5αOH_AAQ56240.2.fasta\|Q6WG30\|T5H_TAXCU | chr9 | 74973367 | 74974270 | - |
| KI387_028856 | Tyu0060200.1 | Taxadiene 5nalpha hydroxylase (Fragment) | | T5αOH_AAQ56240.2.fasta\|Q6WG30\|T5H_TAXCU | chr9 | 74614741 | 74615483 | + |
| KI387_028837 | Tyu0060320.1 | Taxadiene 5nalpha hydroxylase (Fragment) | | T5αOH_AAQ56240.2.fasta\|Q6WG30\|T5H_TAXCU | chr9 | 70257075 | 70259304 | + |
| KI387_031460 | Tyu0086220.1 | Taxane 14b-hydroxylase | | T14βOH_AAO66199.1.fasta\|Q84KI1\|T14H_TAXCU | chr9 | 757919766 | 757921392 | - |
| KI387_010542 | Tyu0102230.1 | 5-alpha-taxadienol-10-beta-hydroxylase | | T10βOH-CYP725A1_AAK00946.1.fasta\|Q9AXM6\|T10H_TAXCU | chr3 | 758361989 | 758363820 | - |
| KI387_026055 | Tyu0114050.1 | CYP728Q13 | | T5αOH_AAQ56240.2.fasta\|Q6WG30\|T5H_TAXCU | chr8 | 137194226 | 137194713 | - |
| KI387_028755 | Tyu0130930.1 | Taxadiene 5nalpha hydroxylase (Fragment) | | T5αOH_AAQ56240.2.fasta\|Q6WG30\|T5H_TAXCU | chr9 | 49866964 | 49867451 | - |
| KI387_011020 | Tyu0131540.1 | CYP725A22 | | T10βOH-CYP725A1_AAK00946.1.fasta\|Q9AXM6\|T10H_TAXCU | chr3 | 864379147 | 864379759 | + |
| KI387_037536 | Tyu0162980.1 | CYP725A18+CYP725A20+T5H | | T13αOH_AAL23619.1.fasta\|Q8W4T9\|T13H_TAXCU | chr11 | 549738522 | 549741575 | - |
| KI387_028549 | Tyu0169960.1 | 13-alpha-hydroxylase | | T13αOH_AAL23619.1.fasta\|Q8W4T9\|T13H_TAXCU | chr9 | 19994272 | 19996422 | + |
| KI387_023189 | Tyu0175670.1 | taxadiene 5-alpha hydroxylase isoform X1 | | T10βOH-CYP725A1_AAK00946.1.fasta\|Q9AXM6\|T10H_TAXCU | chr7 | 184026710 | 184028529 | + |
| KI387_026048 | Tyu0175680.1 | Uncharacterized | | T10βOH-CYP725A1_AAK00946.1.fasta\|Q9AXM6\|T10H_TAXCU | chr8 | 136724962 | 136726994 | + |
| KI387_031200 | Tyu0211500.1 | CYP725A23+CYP725A20+T5H | | T5αOH_AAQ56240.2.fasta\|Q6WG30\|T5H_TAXCU | chr9 | 719219992 | 719222026 | - |
| KI387_028803 | Tyu0234990.1 | Taxadiene 5nalpha hydroxylase (Fragment) | | T5αOH_AAQ56240.2.fasta\|Q6WG30\|T5H_TAXCU | chr9 | 55503341 | 55505492 | + |
| KI387_030680 | Tyu0235030.1 | CYP725A18 | | T13αOH_AAL23619.1.fasta\|Q8W4T9\|T13H_TAXCU | chr9 | 618350012 | 618352118 | - |
| KI387_028798 | Tyu0235040.1 | 5-alpha-taxadienol-10-beta-hydroxylase | | T10βOH-CYP725A1_AAK00946.1.fasta\|Q9AXM6\|T10H_TAXCU | chr9 | 55305161 | 55306743 | + |
| KI387_026111 | Tyu0244550.1 |  | | T5αOH_AAQ56240.2.fasta\|Q6WG30\|T5H_TAXCU | chr8 | 146405913 | 146408340 | - |
| KI387_021617 | Tyu0288350.1 | CYP725B1 | | T5αOH_AAQ56240.2.fasta\|Q6WG30\|T5H_TAXCU | chr6 | 805979865 | 805981560 | + |
| KI387_028799 | Tyu0342110.1 | CYP725A18 | | T5αOH_AAQ56240.2.fasta\|Q6WG30\|T5H_TAXCU | chr9 | 55325866 | 55327835 | + |
| KI387_028800 | Tyu0342120.1 | Taxadiene 5nalpha hydroxylase (Fragment) | | T5αOH_AAQ56240.2.fasta\|Q6WG30\|T5H_TAXCU | chr9 | 55349630 | 55350537 | - |
| KI387_027700 | Tyu0348610.1 | Taxadiene 5nalpha hydroxylase (Fragment) | | T5αOH_AAQ56240.2.fasta\|Q6WG30\|T5H_TAXCU | chr8 | 646405375 | 646409411 | - |

# Table S3 Categories of enzymes in Taxol biosynthetic pathway.

| **Gene Name** | **Gene ID** | **Note** |
| --- | --- | --- |
| CoA Ligase-3 | KI387_028426 | cluster I |
| CoA Ligase-2 | KI387_028438 |  |
| T10βOH_like_13 | KI387_028495 |  |
| T13αOH-3 | KI387_028549 |  |
| T13αOH-2 | KI387_028567 |  |
| T7βOH-1 | KI387_028597 |  |
| T5αOH_like_6 | KI387_028598 |  |
| T2αOH-1 | KI387_028599 |  |
| T10βOH-1 | KI387_028600 |  |
| T10βOH-2 | KI387_028601 |  |
| T5αOH-1 | KI387_028755 |  |
| T10βOH_like_14 | KI387_028798 |  |
| T5αOH_like_7 | KI387_028799 |  |
| T5αOH-4 | KI387_028800 |  |
| TXS-2 | KI387_028802 |  |
| T5αOH-3 | KI387_028803 |  |
| T5αOH_like_8 | KI387_028837 |  |
| TXS-1 | KI387_028840 |  |
| T5αOH_like_9 | KI387_028856 |  |
| T5αOH_like_10 | KI387_028858 |  |
| CoA Ligase-4 | KI387_028882 |  |
| T10βOH_like_15 | KI387_012225 | cluster II |
| T5αOH_like_11 | KI387_030679 |  |
| T13αOH_like_1 | KI387_030680 |  |
| T10βOH_like_16 | KI387_030686 |  |
| T10βOH_like_17 | KI387_030687 |  |
| T10βOH_like_18 | KI387_030692 |  |
| T10βOH_like_19 | KI387_030697 |  |
| T5αOH_like_12 | KI387_030698 |  |
| T10βOH_like_20 | KI387_030703 |  |
| TAT-1 | KI387_030974 |  |
| T5αOH_like_13 | KI387_031200 |  |
| T14βOH-1 | KI387_031460 |  |
| T10βOH_like_21 | KI387_031461 |  |
| GGPPS-1 | KI387_029317 |  |
| TBT-2 | KI387_000888 |  |
| BAPT-1 | KI387_000913 |  |
| TBT-3 | KI387_000915 |  |
| TBT-4 | KI387_000939 |  |
| TBT-1 | KI387_001001 |  |
| T10βOH_like_1 | KI387_001129 |  |
| T10βOH_like_2 | KI387_001515 |  |
| BAPT-3 | KI387_003225 |  |
| BAPT-2 | KI387_005354 |  |
| TB506-1 | KI387_010353 |  |
| T10βOH_like_3 | KI387_010542 |  |
| GGPPS-4 | KI387_010989 |  |
| T10βOH_like_4 | KI387_011020 |  |
| TB506-2 | KI387_011716 |  |
| T13αOH-1 | KI387_015265 |  |
| T5αOH_like_1 | KI387_021617 |  |
| PAM-1 | KI387_022619 |  |
| T10βOH_like_6 | KI387_023119 |  |
| T5αOH_like_2 | KI387_023122 |  |
| T7βOH_like_1 | KI387_023139 |  |
| T10βOH_like_7 | KI387_023189 |  |
| T10βOH_like_8 | KI387_024704 |  |
| T10βOH_like_9 | KI387_024864 |  |
| T10βOH_like_10 | KI387_024865 |  |
| DBTNBT-1 | KI387_025350 |  |
| BAPT-4 | KI387_025352 |  |
| T10βOH_like_11 | KI387_026048 |  |
| T5αOH_like_3 | KI387_026055 |  |
| T10βOH_like_12 | KI387_026089 |  |
| T5αOH_like_4 | KI387_026111 |  |
| T5αOH_like_5 | KI387_027700 |  |
| GGPPS-2 | KI387_027794 |  |
| T10βOH_like_15 | KI387_030673 |  |
| DBTNBT-2 | KI387_034705 |  |
| CoA Ligase-1 | KI387_035235 |  |
| T13αOH_like_2 | KI387_037536 |  |

# Table S4 Identifications of miRNAs in *Taxus chinensis*.

| MiRNA name | Chromosome | Hairpin start | Hairpin end | Strand | MiRNA start | MiRNA end | MiRNA* start | MiRNA* end | MiRNA* detected | TPTM |
| --- | --- | --- | --- | --- | --- | --- | --- | --- | --- | --- |
| tch-miR166a | chr1 | 1043190318 | 1043190443 | + | 1043190403 | 1043190423 | 1043190338 | 1043190358 | YES | 199480 |
| tch-miR166b | chr1 | 1043190800 | 1043190908 | + | 1043190868 | 1043190888 | 1043190820 | 1043190840 | YES | 303590 |
| tch-miR2118a | chr1 | 129323290 | 129323398 | - | 129323310 | 129323332 | 129323357 | 129323378 | NO | 20 |
| tch-miR414 | chr1 | 150681804 | 150681933 | - | 150681893 | 150681913 | 150681824 | 150681847 | NO | 100 |
| tch-miR482a | chr1 | 229341611 | 229341718 | + | 229341678 | 229341698 | 229341631 | 229341651 | NO | 310 |
| tch-miR482b | chr1 | 229410073 | 229410180 | + | 229410140 | 229410160 | 229410093 | 229410113 | YES | 10150 |
| tch-miR11459 | chr1 | 304516754 | 304516862 | + | 304516774 | 304516794 | 304516822 | 304516842 | YES | 394110 |
| tch-miR168a | chr1 | 731717499 | 731717646 | + | 731717607 | 731717627 | 731717519 | 731717539 | YES | 3020 |
| tch-miR477a | chr1 | 809464607 | 809464716 | - | 809464676 | 809464696 | 809464627 | 809464647 | NO | 360 |
| tch-miR4344 | chr1 | 909143558 | 909143757 | + | 909143578 | 909143598 | 909143717 | 909143737 | NO | 750 |
| tch-miR529b | chr1 | 914939483 | 914939613 | - | 914939573 | 914939593 | 914939503 | 914939522 | YES | 260 |
| tch-miR529c | chr1 | 915345238 | 915345339 | - | 915345299 | 915345319 | 915345258 | 915345277 | YES | 90 |
| tch-miR894a | chr1 | 980456507 | 980456595 | - | 980456556 | 980456575 | 980456527 | 980456546 | NO | 20 |
| tch-miR7484.1 | chr1 | 567826262 | 567826396 | + | 567826356 | 567826376 | 567826282 | 567826302 | YES | 150 |
| tch-miR3627.1 | chr1 | 570210032 | 570210133 | + | 570210093 | 570210113 | 570210052 | 570210073 | YES | 8950 |
| tch-miR952.1 | chr1 | 944825038 | 944825149 | - | 944825108 | 944825129 | 944825058 | 944825078 | YES | 86310 |
| tch-miR396g.1 | chr1 | 1007932016 | 1007932123 | - | 1007932083 | 1007932103 | 1007932036 | 1007932056 | YES | 150 |
| tch-miRN1 | chr1 | 86959919 | 86960038 | + | 86959998 | 86960018 | 86959939 | 86959959 | YES | 560 |
| tch-miRN2 | chr1 | 120029704 | 120029787 | + | 120029724 | 120029744 | 120029747 | 120029767 | YES | 420 |
| tch-miRN3 | chr1 | 145494750 | 145494869 | - | 145494770 | 145494790 | 145494829 | 145494849 | YES | 130 |
| tch-miRN4a | chr1 | 146398809 | 146398922 | + | 146398881 | 146398902 | 146398829 | 146398849 | YES | 9830 |
| tch-miRN4b | chr1 | 146421910 | 146422023 | + | 146421982 | 146422003 | 146421930 | 146421950 | YES | 740 |
| tch-miRN5 | chr1 | 186865539 | 186865654 | + | 186865618 | 186865638 | 186865559 | 186865579 | YES | 60 |
| tch-miRN6 | chr1 | 228530368 | 228530481 | - | 228530440 | 228530461 | 228530388 | 228530408 | YES | 4410 |
| tch-miRN7 | chr1 | 273936092 | 273936246 | - | 273936206 | 273936226 | 273936112 | 273936132 | YES | 1180 |
| tch-miRN8 | chr1 | 274156505 | 274156609 | - | 274156589 | 274156609 | 274156525 | 274156545 | YES | 340 |
| tch-miRN9 | chr1 | 274161937 | 274162113 | - | 274161957 | 274161977 | 274162073 | 274162093 | YES | 5050 |
| tch-miRN10 | chr1 | 294532150 | 294532261 | + | 294532170 | 294532190 | 294532221 | 294532241 | YES | 1020 |
| tch-miRN11 | chr1 | 323217313 | 323217473 | + | 323217433 | 323217453 | 323217333 | 323217353 | YES | 50 |
| tch-miRN12 | chr1 | 339825128 | 339825331 | + | 339825148 | 339825168 | 339825291 | 339825311 | YES | 161410 |
| tch-miRN13 | chr1 | 340356849 | 340356969 | - | 340356929 | 340356949 | 340356869 | 340356889 | YES | 790 |
| tch-miRN14 | chr1 | 366842719 | 366842834 | - | 366842794 | 366842814 | 366842739 | 366842759 | YES | 70 |
| tch-miRN15 | chr1 | 522969345 | 522969458 | - | 522969365 | 522969385 | 522969418 | 522969438 | YES | 440 |
| tch-miRN16 | chr1 | 686402874 | 686403050 | + | 686402894 | 686402915 | 686403009 | 686403030 | YES | 20730 |
| tch-miRN17 | chr1 | 728043650 | 728043797 | + | 728043757 | 728043777 | 728043670 | 728043690 | YES | 1420 |
| tch-miRN18 | chr1 | 750705943 | 750706062 | - | 750706022 | 750706042 | 750705963 | 750705983 | YES | 30 |
| tch-miRN19a | chr1 | 805516906 | 805517048 | - | 805517008 | 805517028 | 805516926 | 805516946 | YES | 810 |
| tch-miRN20 | chr1 | 808988652 | 808988757 | + | 808988717 | 808988737 | 808988672 | 808988692 | YES | 1810 |
| tch-miRN21 | chr1 | 898241228 | 898241357 | + | 898241248 | 898241268 | 898241317 | 898241337 | YES | 2930 |
| tch-miRN22 | chr1 | 908776041 | 908776161 | + | 908776121 | 908776141 | 908776061 | 908776081 | YES | 260 |
| tch-miRN23 | chr1 | 918598232 | 918598351 | + | 918598311 | 918598331 | 918598252 | 918598272 | YES | 70 |
| tch-miRN24 | chr1 | 942811037 | 942811171 | - | 942811131 | 942811151 | 942811057 | 942811078 | YES | 52320 |
| tch-miRN25 | chr1 | 953286834 | 953286955 | - | 953286854 | 953286874 | 953286915 | 953286935 | YES | 70 |
| tch-miRN26 | chr1 | 1018514491 | 1018514610 | + | 1018514570 | 1018514590 | 1018514511 | 1018514531 | YES | 290 |
| tch-miR160a | chr10 | 209412682 | 209412802 | + | 209412762 | 209412782 | 209412702 | 209412722 | YES | 1290 |
| tch-miR169a | chr10 | 541539870 | 541539993 | - | 541539953 | 541539973 | 541539890 | 541539910 | NO | 20110 |
| tch-miR169b | chr10 | 541638627 | 541638751 | + | 541638647 | 541638667 | 541638710 | 541638731 | YES | 4070 |
| tch-miR169c | chr10 | 542283434 | 542283559 | - | 542283519 | 542283539 | 542283454 | 542283474 | NO | 1200 |
| tch-miR169d | chr10 | 542484450 | 542484577 | + | 542484470 | 542484490 | 542484537 | 542484557 | NO | 150 |
| tch-miR169e | chr10 | 542929812 | 542929944 | + | 542929832 | 542929852 | 542929904 | 542929924 | NO | 17560 |
| tch-miR169f | chr10 | 572928133 | 572928247 | + | 572928153 | 572928173 | 572928207 | 572928227 | YES | 77520 |
| tch-miR169g | chr10 | 574038390 | 574038504 | - | 574038463 | 574038484 | 574038410 | 574038431 | NO | 60 |
| tch-miR5780 | chr10 | 7253729 | 7253842 | - | 7253803 | 7253822 | 7253749 | 7253768 | NO | 40 |
| tch-miR7539.1 | chr10 | 343682010 | 343682104 | + | 343682030 | 343682050 | 343682064 | 343682084 | YES | 15340 |
| tch-miR169n.1 | chr10 | 576403278 | 576403406 | + | 576403366 | 576403386 | 576403298 | 576403318 | YES | 270 |
| tch-miRN177 | chr10 | 48903096 | 48903242 | + | 48903116 | 48903136 | 48903202 | 48903222 | YES | 3570 |
| tch-miRN44b | chr10 | 51430126 | 51430267 | - | 51430146 | 51430166 | 51430227 | 51430247 | YES | 780 |
| tch-miRN178 | chr10 | 146755967 | 146756090 | - | 146756050 | 146756070 | 146755987 | 146756007 | YES | 230 |
| tch-miRN179 | chr10 | 170998791 | 170998989 | + | 170998811 | 170998831 | 170998949 | 170998969 | YES | 70 |
| tch-miRN180a | chr10 | 204963290 | 204963433 | + | 204963392 | 204963413 | 204963310 | 204963331 | YES | 583690 |
| tch-miRN180b | chr10 | 204967260 | 204967400 | + | 204967359 | 204967380 | 204967280 | 204967301 | YES | 17220 |
| tch-miRN181 | chr10 | 212694443 | 212694562 | - | 212694463 | 212694483 | 212694522 | 212694542 | YES | 270 |
| tch-miRN182 | chr10 | 230694905 | 230695023 | - | 230694925 | 230694945 | 230694980 | 230695003 | YES | 13150 |
| tch-miRN183 | chr10 | 231872245 | 231872389 | - | 231872265 | 231872285 | 231872349 | 231872369 | YES | 20440 |
| tch-miRN184 | chr10 | 234808602 | 234808721 | - | 234808622 | 234808642 | 234808681 | 234808701 | YES | 30 |
| tch-miRN185 | chr10 | 236334212 | 236334332 | - | 236334232 | 236334252 | 236334291 | 236334312 | YES | 1640 |
| tch-miRN186 | chr10 | 252560812 | 252560933 | + | 252560893 | 252560913 | 252560832 | 252560852 | YES | 120 |
| tch-miRN187 | chr10 | 393530963 | 393531180 | + | 393531140 | 393531160 | 393530983 | 393531003 | YES | 112100 |
| tch-miRN188 | chr10 | 405630651 | 405630785 | - | 405630671 | 405630691 | 405630745 | 405630765 | YES | 330 |
| tch-miRN189 | chr10 | 422203476 | 422203598 | + | 422203562 | 422203582 | 422203496 | 422203516 | YES | 5620 |
| tch-miRN190a | chr10 | 465922662 | 465922817 | + | 465922682 | 465922702 | 465922777 | 465922797 | YES | 4310 |
| tch-miRN190b | chr10 | 466029181 | 466029336 | + | 466029201 | 466029221 | 466029296 | 466029316 | YES | 4510 |
| tch-miRN190c | chr10 | 466187371 | 466187517 | + | 466187391 | 466187411 | 466187477 | 466187497 | YES | 2973760 |
| tch-miRN191 | chr10 | 473594886 | 473595044 | + | 473594906 | 473594926 | 473595004 | 473595024 | YES | 370 |
| tch-miRN192 | chr10 | 576989981 | 576990083 | + | 576990001 | 576990021 | 576990043 | 576990063 | YES | 10070 |
| tch-miRN193 | chr10 | 580949744 | 580949870 | + | 580949764 | 580949785 | 580949830 | 580949850 | YES | 110770 |
| tch-miRN194 | chr10 | 622585197 | 622585497 | - | 622585217 | 622585237 | 622585457 | 622585477 | YES | 82890 |
| tch-miRN195 | chr10 | 627616534 | 627616653 | + | 627616613 | 627616633 | 627616554 | 627616574 | YES | 270 |
| tch-miRN196 | chr10 | 645010579 | 645010723 | + | 645010683 | 645010703 | 645010599 | 645010619 | YES | 240 |
| tch-miR399a | chr11 | 150704749 | 150704878 | - | 150704769 | 150704789 | 150704838 | 150704858 | YES | 520 |
| tch-miR399b | chr11 | 151339164 | 151339283 | + | 151339243 | 151339263 | 151339184 | 151339204 | YES | 1450 |
| tch-miR8762 | chr11 | 152844776 | 152844937 | - | 152844897 | 152844917 | 152844796 | 152844816 | NO | 40 |
| tch-miR160b | chr11 | 239663386 | 239663507 | - | 239663406 | 239663426 | 239663467 | 239663487 | YES | 200 |
| tch-miR11034 | chr11 | 397210552 | 397210642 | - | 397210605 | 397210625 | 397210572 | 397210594 | NO | 20 |
| tch-miR390a | chr11 | 447279824 | 447279939 | + | 447279844 | 447279864 | 447279899 | 447279919 | YES | 367170 |
| tch-miR395a | chr11 | 504348262 | 504348375 | - | 504348282 | 504348302 | 504348335 | 504348355 | YES | 20980 |
| tch-miR395b | chr11 | 504915582 | 504915693 | - | 504915602 | 504915622 | 504915653 | 504915673 | YES | 11780 |
| tch-miR395c | chr11 | 504952133 | 504952235 | + | 504952196 | 504952215 | 504952153 | 504952173 | YES | 68620 |
| tch-miR395d | chr11 | 506461363 | 506461485 | + | 506461446 | 506461465 | 506461383 | 506461402 | NO | 1410 |
| tch-miR164a | chr11 | 593721795 | 593721911 | + | 593721815 | 593721835 | 593721871 | 593721891 | YES | 858620 |
| tch-miR11109.1 | chr11 | 483088588 | 483088702 | - | 483088663 | 483088682 | 483088608 | 483088628 | YES | 1750 |
| tch-miR1536.1 | chr11 | 616768613 | 616768720 | - | 616768633 | 616768653 | 616768680 | 616768700 | YES | 2090 |
| tch-miRN197 | chr11 | 5989329 | 5989467 | - | 5989427 | 5989447 | 5989349 | 5989369 | YES | 1900 |
| tch-miRN198 | chr11 | 60548363 | 60548488 | + | 60548383 | 60548403 | 60548448 | 60548468 | YES | 14430 |
| tch-miRN199 | chr11 | 111541919 | 111542044 | - | 111541939 | 111541960 | 111542003 | 111542024 | YES | 51080 |
| tch-miRN200 | chr11 | 152415817 | 152416089 | - | 152415837 | 152415858 | 152416048 | 152416069 | YES | 39330 |
| tch-miRN201 | chr11 | 169100920 | 169101030 | - | 169100990 | 169101010 | 169100940 | 169100960 | YES | 130 |
| tch-miRN202 | chr11 | 228319546 | 228319629 | + | 228319566 | 228319586 | 228319589 | 228319609 | YES | 60 |
| tch-miRN53b | chr11 | 235441502 | 235441597 | + | 235441557 | 235441577 | 235441522 | 235441542 | YES | 280 |
| tch-miRN203 | chr11 | 240080050 | 240080183 | + | 240080143 | 240080163 | 240080070 | 240080090 | YES | 970 |
| tch-miRN204 | chr11 | 326546289 | 326546427 | - | 326546309 | 326546329 | 326546387 | 326546407 | YES | 250 |
| tch-miRN205 | chr11 | 421048858 | 421049007 | - | 421048878 | 421048898 | 421048967 | 421048987 | YES | 470 |
| tch-miRN206 | chr11 | 479577137 | 479577264 | + | 479577224 | 479577244 | 479577157 | 479577177 | YES | 980 |
| tch-miRN73b | chr11 | 483088758 | 483088878 | - | 483088839 | 483088858 | 483088778 | 483088798 | YES | 311140 |
| tch-miRN73d | chr11 | 483343536 | 483343656 | - | 483343556 | 483343575 | 483343618 | 483343636 | YES | 348490 |
| tch-miRN73c | chr11 | 483594544 | 483594653 | - | 483594613 | 483594633 | 483594564 | 483594584 | YES | 3160 |
| tch-miRN207 | chr11 | 597104057 | 597104176 | - | 597104077 | 597104097 | 597104136 | 597104156 | YES | 270 |
| tch-miRN208 | chr11 | 600701290 | 600701410 | + | 600701369 | 600701390 | 600701310 | 600701330 | YES | 500 |
| tch-miRN209 | chr11 | 616731516 | 616731869 | - | 616731536 | 616731557 | 616731828 | 616731849 | YES | 44470 |
| tch-miR11309 | chr12 | 130329677 | 130329799 | + | 130329697 | 130329717 | 130329760 | 130329779 | NO | 1840 |
| tch-miR529a | chr12 | 173774791 | 173774917 | - | 173774877 | 173774897 | 173774811 | 173774831 | NO | 790 |
| tch-miR169h | chr12 | 185661363 | 185661497 | + | 185661383 | 185661403 | 185661457 | 185661477 | YES | 15620 |
| tch-miR11485 | chr12 | 252163084 | 252163209 | - | 252163169 | 252163189 | 252163104 | 252163124 | NO | 480 |
| tch-miR390b | chr12 | 396745619 | 396745726 | - | 396745686 | 396745706 | 396745639 | 396745659 | NO | 950 |
| tch-miR1314 | chr12 | 49366365 | 49366471 | - | 49366385 | 49366406 | 49366431 | 49366451 | NO | 160 |
| tch-miR11027.1 | chr12 | 45790120 | 45790256 | + | 45790216 | 45790236 | 45790140 | 45790160 | YES | 230 |
| tch-miR396f.1 | chr12 | 111096181 | 111096314 | + | 111096201 | 111096221 | 111096274 | 111096294 | YES | 960 |
| tch-miR8020.1 | chr12 | 254106521 | 254106621 | - | 254106581 | 254106601 | 254106541 | 254106561 | YES | 3970 |
| tch-miRN210 | chr12 | 47556095 | 47556231 | - | 47556115 | 47556135 | 47556191 | 47556211 | YES | 180 |
| tch-miRN176b | chr12 | 56150745 | 56150862 | - | 56150822 | 56150842 | 56150765 | 56150785 | YES | 1170 |
| tch-miRN211 | chr12 | 158034673 | 158034795 | + | 158034693 | 158034713 | 158034755 | 158034775 | YES | 190 |
| tch-miRN212 | chr12 | 195025817 | 195025938 | + | 195025898 | 195025918 | 195025837 | 195025857 | YES | 50 |
| tch-miRN213 | chr12 | 203982745 | 203982862 | - | 203982765 | 203982785 | 203982822 | 203982842 | YES | 100 |
| tch-miRN214 | chr12 | 234103857 | 234103976 | - | 234103877 | 234103897 | 234103936 | 234103956 | YES | 110 |
| tch-miRN215 | chr12 | 239252065 | 239252214 | + | 239252085 | 239252105 | 239252174 | 239252194 | YES | 160 |
| tch-miRN216 | chr12 | 263477526 | 263477642 | - | 263477601 | 263477622 | 263477546 | 263477566 | YES | 60 |
| tch-miRN112b | chr12 | 384015829 | 384015949 | - | 384015849 | 384015869 | 384015909 | 384015929 | YES | 250 |
| tch-miR6273 | chr2 | 211805035 | 211805129 | + | 211805090 | 211805109 | 211805055 | 211805073 | NO | 30 |
| tch-miR1030 | chr2 | 282792714 | 282792813 | - | 282792773 | 282792793 | 282792734 | 282792754 | YES | 1850 |
| tch-miR11411 | chr2 | 302475428 | 302475609 | + | 302475568 | 302475589 | 302475448 | 302475468 | YES | 3120 |
| tch-miR394 | chr2 | 841859434 | 841859547 | + | 841859454 | 841859473 | 841859507 | 841859527 | NO | 650 |
| tch-miR393 | chr2 | 896625824 | 896625923 | + | 896625844 | 896625864 | 896625884 | 896625903 | NO | 1810 |
| tch-miR166c | chr2 | 907882824 | 907882939 | + | 907882899 | 907882919 | 907882844 | 907882864 | YES | 19350 |
| tch-miR319a | chr2 | 927880031 | 927880131 | - | 927880051 | 927880070 | 927880092 | 927880111 | NO | 6280 |
| tch-miR169i | chr2 | 958194277 | 958194375 | - | 958194336 | 958194355 | 958194297 | 958194317 | NO | 20 |
| tch-miR8608.1 | chr2 | 7793894 | 7794041 | - | 7793914 | 7793934 | 7794001 | 7794021 | YES | 470 |
| tch-miR11314.1 | chr2 | 30621839 | 30621926 | + | 30621886 | 30621906 | 30621859 | 30621882 | YES | 50 |
| tch-miR2602.1 | chr2 | 49757219 | 49757382 | + | 49757239 | 49757259 | 49757342 | 49757362 | YES | 1810 |
| tch-miR2607.1 | chr2 | 514765098 | 514765293 | - | 514765118 | 514765138 | 514765253 | 514765273 | YES | 7760 |
| tch-miR11431.1 | chr2 | 696424074 | 696424239 | - | 696424199 | 696424219 | 696424094 | 696424114 | YES | 4410 |
| tch-miRN27 | chr2 | 2748862 | 2749011 | - | 2748971 | 2748991 | 2748882 | 2748901 | YES | 31760 |
| tch-miRN28 | chr2 | 42165893 | 42165998 | + | 42165913 | 42165933 | 42165958 | 42165978 | YES | 40 |
| tch-miRN29 | chr2 | 97078821 | 97078945 | + | 97078841 | 97078861 | 97078905 | 97078925 | YES | 17040 |
| tch-miRN30 | chr2 | 142503079 | 142503217 | + | 142503177 | 142503197 | 142503099 | 142503119 | YES | 680 |
| tch-miRN31 | chr2 | 165563080 | 165563236 | - | 165563196 | 165563216 | 165563100 | 165563120 | YES | 150 |
| tch-miRN32 | chr2 | 196061987 | 196062116 | - | 196062007 | 196062027 | 196062076 | 196062096 | YES | 4110 |
| tch-miRN33 | chr2 | 207685448 | 207685602 | + | 207685561 | 207685582 | 207685468 | 207685489 | YES | 28550 |
| tch-miRN34 | chr2 | 242206342 | 242206479 | + | 242206439 | 242206459 | 242206362 | 242206382 | YES | 2610 |
| tch-miRN35 | chr2 | 252747064 | 252747175 | - | 252747084 | 252747104 | 252747135 | 252747155 | YES | 100 |
| tch-miRN36 | chr2 | 282876690 | 282876796 | - | 282876710 | 282876730 | 282876756 | 282876776 | YES | 760 |
| tch-miRN37 | chr2 | 317043523 | 317043659 | - | 317043619 | 317043639 | 317043543 | 317043563 | YES | 560 |
| tch-miRN38 | chr2 | 350015618 | 350015739 | - | 350015638 | 350015658 | 350015699 | 350015719 | YES | 60 |
| tch-miRN39a | chr2 | 379133907 | 379134022 | - | 379133927 | 379133947 | 379133982 | 379134002 | YES | 80 |
| tch-miRN40a | chr2 | 379224711 | 379224810 | + | 379224781 | 379224801 | 379224731 | 379224751 | YES | 50 |
| tch-miRN41 | chr2 | 393379938 | 393380062 | - | 393379958 | 393379978 | 393380022 | 393380042 | YES | 160 |
| tch-miRN42 | chr2 | 497275606 | 497275744 | + | 497275626 | 497275645 | 497275705 | 497275724 | YES | 240 |
| tch-miRN43 | chr2 | 499873961 | 499874076 | + | 499873981 | 499874001 | 499874036 | 499874056 | YES | 1690 |
| tch-miRN44a | chr2 | 500077651 | 500077763 | + | 500077723 | 500077743 | 500077671 | 500077691 | YES | 120 |
| tch-miRN40d | chr2 | 508240477 | 508240596 | - | 508240497 | 508240517 | 508240556 | 508240576 | YES | 540 |
| tch-miRN45 | chr2 | 603114024 | 603114145 | - | 603114105 | 603114125 | 603114044 | 603114064 | YES | 180 |
| tch-miRN46 | chr2 | 667536471 | 667536598 | + | 667536491 | 667536512 | 667536558 | 667536578 | YES | 26130 |
| tch-miRN47 | chr2 | 676473500 | 676473619 | + | 676473579 | 676473599 | 676473520 | 676473540 | YES | 390 |
| tch-miRN48 | chr2 | 717938344 | 717938464 | - | 717938364 | 717938384 | 717938424 | 717938444 | YES | 1700 |
| tch-miRN49 | chr2 | 751105611 | 751105728 | - | 751105687 | 751105708 | 751105631 | 751105652 | YES | 249270 |
| tch-miRN50 | chr2 | 780239589 | 780239700 | - | 780239609 | 780239630 | 780239659 | 780239680 | YES | 307910 |
| tch-miRN51 | chr2 | 787662661 | 787662781 | + | 787662741 | 787662761 | 787662681 | 787662701 | YES | 1700 |
| tch-miRN52a | chr2 | 849792274 | 849792385 | - | 849792345 | 849792365 | 849792294 | 849792314 | YES | 26410 |
| tch-miRN52c | chr2 | 850020966 | 850021077 | - | 850021037 | 850021057 | 850020986 | 850021006 | YES | 26570 |
| tch-miRN52b | chr2 | 850027436 | 850027547 | - | 850027507 | 850027527 | 850027456 | 850027476 | YES | 27490 |
| tch-miRN53a | chr2 | 853862970 | 853863045 | + | 853863025 | 853863045 | 853862990 | 853863010 | YES | 130 |
| tch-miRN40b | chr2 | 877955092 | 877955211 | - | 877955171 | 877955191 | 877955112 | 877955132 | YES | 80 |
| tch-miRN54 | chr2 | 921805624 | 921805800 | + | 921805644 | 921805664 | 921805760 | 921805780 | YES | 340 |
| tch-miRN55 | chr2 | 940585933 | 940586054 | - | 940585953 | 940585973 | 940586014 | 940586034 | YES | 2540 |
| tch-miRN56 | chr2 | 954023243 | 954023389 | + | 954023349 | 954023369 | 954023263 | 954023283 | YES | 780 |
| tch-miRN57 | chr2 | 956721454 | 956721573 | - | 956721474 | 956721494 | 956721533 | 956721553 | YES | 290 |
| tch-miR9722 | chr3 | 147950265 | 147950344 | - | 147950285 | 147950304 | 147950303 | 147950324 | NO | 20 |
| tch-miR162 | chr3 | 148609880 | 148610018 | + | 148609978 | 148609998 | 148609900 | 148609920 | YES | 196190 |
| tch-miR2118b | chr3 | 161272320 | 161272433 | - | 161272340 | 161272361 | 161272393 | 161272413 | YES | 134360 |
| tch-miR482c | chr3 | 215699646 | 215699754 | - | 215699666 | 215699687 | 215699714 | 215699734 | YES | 3957410 |
| tch-miR482d | chr3 | 215896402 | 215896527 | - | 215896422 | 215896443 | 215896487 | 215896507 | YES | 1070140 |
| tch-miR482e | chr3 | 216514795 | 216514918 | - | 216514815 | 216514836 | 216514878 | 216514898 | YES | 1101680 |
| tch-miR396a | chr3 | 219618823 | 219618990 | + | 219618951 | 219618970 | 219618843 | 219618862 | YES | 14290 |
| tch-miR396b | chr3 | 220055776 | 220055910 | + | 220055796 | 220055816 | 220055870 | 220055890 | YES | 102640 |
| tch-miR8175a | chr3 | 302518003 | 302518139 | - | 302518023 | 302518043 | 302518099 | 302518119 | NO | 270 |
| tch-miR472a | chr3 | 617091838 | 617091940 | - | 617091858 | 617091879 | 617091900 | 617091920 | YES | 2691070 |
| tch-miR482f | chr3 | 617706698 | 617706795 | + | 617706754 | 617706775 | 617706718 | 617706740 | YES | 56250 |
| tch-miR164b | chr3 | 670880506 | 670880606 | - | 670880566 | 670880586 | 670880526 | 670880546 | YES | 6170 |
| tch-miR164c | chr3 | 671158991 | 671159105 | - | 671159065 | 671159085 | 671159011 | 671159031 | YES | 518290 |
| tch-miR3693 | chr3 | 734933429 | 734933511 | - | 734933449 | 734933469 | 734933469 | 734933491 | NO | 50 |
| tch-miR482g | chr3 | 773954090 | 773954189 | + | 773954148 | 773954169 | 773954110 | 773954130 | YES | 552750 |
| tch-miR482h | chr3 | 773955216 | 773955317 | + | 773955276 | 773955297 | 773955236 | 773955256 | YES | 169670 |
| tch-miR482i | chr3 | 846118649 | 846118752 | + | 846118711 | 846118732 | 846118669 | 846118689 | NO | 40 |
| tch-miR319b | chr3 | 880359168 | 880359380 | + | 880359341 | 880359360 | 880359188 | 880359207 | NO | 260 |
| tch-miR169j | chr3 | 888649577 | 888649691 | + | 888649597 | 888649617 | 888649651 | 888649671 | YES | 12380 |
| tch-miR159a | chr3 | 938574885 | 938575085 | + | 938575046 | 938575065 | 938574905 | 938574924 | NO | 19490 |
| tch-miR319c | chr3 | 939482728 | 939482940 | + | 939482900 | 939482920 | 939482748 | 939482768 | NO | 160 |
| tch-miR159b | chr3 | 942280874 | 942281086 | - | 942280894 | 942280914 | 942281046 | 942281066 | YES | 224500 |
| tch-miR166h.1 | chr3 | 45719764 | 45719926 | - | 45719784 | 45719804 | 45719886 | 45719906 | YES | 107270 |
| tch-miR11552.1 | chr3 | 236966515 | 236966645 | - | 236966605 | 236966625 | 236966535 | 236966557 | YES | 340 |
| tch-miR821.1 | chr3 | 641429791 | 641429939 | + | 641429899 | 641429919 | 641429811 | 641429831 | YES | 100 |
| tch-miR858.1 | chr3 | 681998966 | 681999082 | + | 681999043 | 681999062 | 681998986 | 681999005 | YES | 7840 |
| tch-miR167b.1 | chr3 | 892772858 | 892772968 | - | 892772928 | 892772948 | 892772878 | 892772898 | YES | 960 |
| tch-miRN58 | chr3 | 112930474 | 112930589 | - | 112930494 | 112930514 | 112930549 | 112930569 | YES | 20450 |
| tch-miRN59 | chr3 | 138943783 | 138943970 | + | 138943929 | 138943950 | 138943803 | 138943824 | YES | 600 |
| tch-miRN60 | chr3 | 150161692 | 150161854 | + | 150161712 | 150161732 | 150161814 | 150161834 | YES | 3650 |
| tch-miRN61 | chr3 | 160167847 | 160167949 | - | 160167909 | 160167929 | 160167867 | 160167889 | YES | 80 |
| tch-miRN62 | chr3 | 208037848 | 208037967 | - | 208037868 | 208037888 | 208037927 | 208037947 | YES | 310 |
| tch-miRN63 | chr3 | 230839242 | 230839361 | + | 230839262 | 230839282 | 230839321 | 230839341 | YES | 1560 |
| tch-miRN64 | chr3 | 386245517 | 386245666 | - | 386245626 | 386245646 | 386245537 | 386245557 | YES | 160 |
| tch-miRN40c | chr3 | 408492881 | 408493000 | - | 408492960 | 408492980 | 408492901 | 408492921 | YES | 330 |
| tch-miRN40f | chr3 | 423198556 | 423198677 | + | 423198637 | 423198657 | 423198576 | 423198596 | YES | 170 |
| tch-miRN65 | chr3 | 576361810 | 576361931 | - | 576361891 | 576361911 | 576361830 | 576361850 | YES | 180 |
| tch-miRN66 | chr3 | 592861667 | 592861810 | + | 592861770 | 592861790 | 592861687 | 592861707 | YES | 650 |
| tch-miRN67 | chr3 | 594082925 | 594083073 | + | 594083032 | 594083053 | 594082945 | 594082966 | YES | 700 |
| tch-miRN68 | chr3 | 594166944 | 594167089 | + | 594166964 | 594166984 | 594167049 | 594167069 | YES | 240 |
| tch-miRN69 | chr3 | 600925346 | 600925468 | - | 600925366 | 600925386 | 600925428 | 600925448 | YES | 450 |
| tch-miRN70 | chr3 | 626655929 | 626656054 | - | 626656013 | 626656034 | 626655949 | 626655969 | YES | 1410 |
| tch-miRN71 | chr3 | 643585433 | 643585618 | - | 643585578 | 643585598 | 643585453 | 643585473 | YES | 180 |
| tch-miRN72 | chr3 | 785386493 | 785386659 | + | 785386513 | 785386533 | 785386619 | 785386639 | YES | 390 |
| tch-miRN73a | chr3 | 786888314 | 786888423 | - | 786888383 | 786888403 | 786888334 | 786888354 | YES | 3450 |
| tch-miRN74 | chr3 | 821889805 | 821889918 | - | 821889825 | 821889845 | 821889878 | 821889898 | YES | 170 |
| tch-miRN39b | chr3 | 861265486 | 861265608 | + | 861265568 | 861265588 | 861265506 | 861265526 | YES | 810 |
| tch-miRN75 | chr3 | 876564463 | 876564582 | + | 876564483 | 876564503 | 876564542 | 876564562 | YES | 1000 |
| tch-miRN76 | chr3 | 889587497 | 889587625 | + | 889587517 | 889587537 | 889587585 | 889587605 | YES | 70 |
| tch-miR535a | chr4 | 197090412 | 197090540 | + | 197090432 | 197090453 | 197090498 | 197090520 | NO | 7290 |
| tch-miR1083 | chr4 | 359409091 | 359409204 | - | 359409165 | 359409184 | 359409111 | 359409130 | YES | 481520 |
| tch-miR894b | chr4 | 453825911 | 453826158 | - | 453826119 | 453826138 | 453825931 | 453825951 | NO | 30 |
| tch-miR2950a | chr4 | 504434349 | 504434465 | - | 504434425 | 504434445 | 504434369 | 504434389 | NO | 110 |
| tch-miR11534 | chr4 | 510574214 | 510574448 | + | 510574234 | 510574253 | 510574409 | 510574428 | NO | 30 |
| tch-miR172 | chr4 | 587113178 | 587113307 | - | 587113267 | 587113287 | 587113198 | 587113218 | YES | 551680 |
| tch-miR395e | chr4 | 675485526 | 675485652 | + | 675485546 | 675485565 | 675485613 | 675485632 | NO | 130 |
| tch-miR395f | chr4 | 675594166 | 675594292 | + | 675594186 | 675594205 | 675594253 | 675594272 | NO | 120 |
| tch-miR395g | chr4 | 675597462 | 675597588 | + | 675597482 | 675597501 | 675597549 | 675597568 | NO | 160 |
| tch-miR395h | chr4 | 675718767 | 675718893 | + | 675718787 | 675718806 | 675718854 | 675718873 | NO | 170 |
| tch-miR395i | chr4 | 675866620 | 675866747 | + | 675866640 | 675866659 | 675866708 | 675866727 | NO | 150 |
| tch-miR169k | chr4 | 738203165 | 738203246 | - | 738203219 | 738203238 | 738203185 | 738203202 | NO | 20 |
| tch-miR1871 | chr4 | 79311244 | 79311485 | - | 79311446 | 79311465 | 79311264 | 79311283 | NO | 20 |
| tch-miR536 | chr4 | 884273059 | 884273176 | + | 884273136 | 884273156 | 884273079 | 884273098 | NO | 20 |
| tch-miR168b | chr4 | 926544245 | 926544374 | - | 926544334 | 926544354 | 926544265 | 926544285 | YES | 476600 |
| tch-miR7980.1 | chr4 | 186479304 | 186479424 | - | 186479324 | 186479344 | 186479384 | 186479404 | YES | 870 |
| tch-miR906a.1 | chr4 | 352215093 | 352215212 | - | 352215113 | 352215133 | 352215172 | 352215192 | YES | 670 |
| tch-miR10980.1 | chr4 | 666680437 | 666680527 | + | 666680495 | 666680515 | 666680457 | 666680477 | YES | 2080 |
| tch-miR7762.1 | chr4 | 713386845 | 713386944 | - | 713386865 | 713386885 | 713386904 | 713386924 | YES | 43890 |
| tch-miR11531.1 | chr4 | 828819010 | 828819136 | - | 828819096 | 828819116 | 828819030 | 828819050 | YES | 380 |
| tch-miRN77 | chr4 | 16327301 | 16327419 | - | 16327382 | 16327403 | 16327321 | 16327342 | YES | 200 |
| tch-miRN78 | chr4 | 115459055 | 115459213 | + | 115459075 | 115459095 | 115459173 | 115459193 | YES | 160 |
| tch-miRN79 | chr4 | 118449170 | 118449275 | + | 118449190 | 118449210 | 118449235 | 118449255 | YES | 50 |
| tch-miRN80 | chr4 | 134898096 | 134898218 | - | 134898178 | 134898198 | 134898116 | 134898136 | YES | 50 |
| tch-miRN40e | chr4 | 159926132 | 159926229 | + | 159926189 | 159926209 | 159926152 | 159926172 | YES | 2430 |
| tch-miRN81 | chr4 | 160559601 | 160559698 | - | 160559672 | 160559692 | 160559621 | 160559641 | YES | 240 |
| tch-miRN82 | chr4 | 192265332 | 192265432 | + | 192265392 | 192265412 | 192265352 | 192265372 | YES | 50 |
| tch-miRN83 | chr4 | 255869664 | 255869785 | - | 255869744 | 255869765 | 255869684 | 255869704 | YES | 1230 |
| tch-miRN84 | chr4 | 261206752 | 261206876 | - | 261206835 | 261206856 | 261206772 | 261206792 | YES | 60 |
| tch-miRN85 | chr4 | 293701696 | 293701827 | + | 293701716 | 293701736 | 293701787 | 293701807 | YES | 1670 |
| tch-miRN86 | chr4 | 307855952 | 307856117 | - | 307855972 | 307855992 | 307856077 | 307856097 | YES | 220 |
| tch-miRN87 | chr4 | 352261859 | 352261981 | - | 352261940 | 352261961 | 352261879 | 352261900 | YES | 100 |
| tch-miRN88 | chr4 | 369255959 | 369256098 | - | 369255979 | 369255999 | 369256058 | 369256078 | YES | 60 |
| tch-miRN89 | chr4 | 385175685 | 385175876 | + | 385175836 | 385175856 | 385175705 | 385175725 | YES | 130 |
| tch-miRN90 | chr4 | 432780179 | 432780298 | - | 432780259 | 432780278 | 432780199 | 432780218 | YES | 2460 |
| tch-miRN91 | chr4 | 502242359 | 502242496 | - | 502242456 | 502242476 | 502242379 | 502242399 | YES | 80 |
| tch-miRN92 | chr4 | 531771850 | 531771967 | + | 531771927 | 531771947 | 531771870 | 531771890 | YES | 15440 |
| tch-miRN93 | chr4 | 544812062 | 544812179 | - | 544812139 | 544812159 | 544812082 | 544812102 | YES | 90 |
| tch-miRN94 | chr4 | 556157351 | 556157513 | + | 556157473 | 556157493 | 556157371 | 556157391 | YES | 1960 |
| tch-miRN40l | chr4 | 597774765 | 597774884 | - | 597774844 | 597774864 | 597774785 | 597774805 | YES | 110 |
| tch-miRN95 | chr4 | 640451923 | 640452046 | + | 640451943 | 640451963 | 640452006 | 640452026 | YES | 240 |
| tch-miRN96a | chr4 | 641928156 | 641928279 | + | 641928176 | 641928196 | 641928239 | 641928259 | YES | 18490 |
| tch-miRN97 | chr4 | 651668017 | 651668138 | + | 651668037 | 651668057 | 651668098 | 651668118 | YES | 60 |
| tch-miRN98 | chr4 | 660618064 | 660618234 | - | 660618084 | 660618104 | 660618193 | 660618214 | YES | 430 |
| tch-miRN99 | chr4 | 661626241 | 661626386 | + | 661626346 | 661626366 | 661626261 | 661626281 | YES | 90 |
| tch-miRN96b | chr4 | 712350652 | 712350775 | - | 712350735 | 712350755 | 712350672 | 712350692 | YES | 17770 |
| tch-miRN100 | chr4 | 723790910 | 723791032 | + | 723790930 | 723790950 | 723790992 | 723791012 | YES | 310 |
| tch-miRN101a | chr4 | 780351683 | 780351783 | + | 780351703 | 780351723 | 780351742 | 780351763 | YES | 100 |
| tch-miRN102 | chr4 | 866273290 | 866273409 | - | 866273310 | 866273330 | 866273369 | 866273389 | YES | 3730 |
| tch-miRN103 | chr4 | 895213711 | 895213836 | - | 895213731 | 895213751 | 895213796 | 895213816 | YES | 1380 |
| tch-miRN104 | chr4 | 896715690 | 896715804 | - | 896715710 | 896715730 | 896715764 | 896715784 | YES | 14000 |
| tch-miRN105 | chr4 | 929776890 | 929777010 | - | 929776970 | 929776990 | 929776910 | 929776930 | YES | 880 |
| tch-miR166d | chr5 | 168697705 | 168697814 | + | 168697774 | 168697794 | 168697725 | 168697745 | YES | 110330 |
| tch-miR169l | chr5 | 231046720 | 231046843 | - | 231046804 | 231046823 | 231046740 | 231046759 | YES | 800 |
| tch-miR166e | chr5 | 346295191 | 346295289 | - | 346295211 | 346295231 | 346295249 | 346295269 | YES | 384470 |
| tch-miR7820 | chr5 | 450557617 | 450557758 | + | 450557718 | 450557738 | 450557637 | 450557657 | NO | 20 |
| tch-miR391 | chr5 | 53546350 | 53546468 | - | 53546428 | 53546448 | 53546370 | 53546390 | YES | 11200 |
| tch-miR5225 | chr5 | 53577795 | 53577894 | - | 53577853 | 53577874 | 53577815 | 53577835 | YES | 460 |
| tch-miR3948 | chr5 | 575479432 | 575479604 | + | 575479452 | 575479472 | 575479565 | 575479584 | NO | 30 |
| tch-miR482j | chr5 | 72662210 | 72662314 | - | 72662230 | 72662251 | 72662274 | 72662294 | YES | 2172780 |
| tch-miR482k | chr5 | 73109814 | 73109914 | + | 73109873 | 73109894 | 73109834 | 73109854 | YES | 129330 |
| tch-miR482l | chr5 | 73235838 | 73235934 | + | 73235893 | 73235914 | 73235858 | 73235878 | YES | 934790 |
| tch-miR529d | chr5 | 774610533 | 774610650 | - | 774610610 | 774610630 | 774610553 | 774610573 | YES | 66770 |
| tch-miR829 | chr5 | 781622983 | 781623107 | + | 781623003 | 781623026 | 781623067 | 781623087 | NO | 160 |
| tch-miR11464 | chr5 | 787803438 | 787803560 | + | 787803458 | 787803478 | 787803520 | 787803540 | NO | 40 |
| tch-miR397a | chr5 | 805623207 | 805623331 | + | 805623227 | 805623247 | 805623291 | 805623311 | YES | 1404240 |
| tch-miR397b | chr5 | 806040709 | 806040837 | - | 806040797 | 806040817 | 806040729 | 806040749 | YES | 434370 |
| tch-miR397c | chr5 | 806178315 | 806178441 | + | 806178335 | 806178355 | 806178401 | 806178421 | YES | 182570 |
| tch-miR397d | chr5 | 806448779 | 806448902 | + | 806448799 | 806448819 | 806448862 | 806448882 | YES | 441480 |
| tch-miR397e | chr5 | 806449054 | 806449178 | + | 806449074 | 806449096 | 806449137 | 806449158 | NO | 15470 |
| tch-miR529e | chr5 | 823885943 | 823886051 | - | 823886011 | 823886031 | 823885963 | 823885983 | NO | 780 |
| tch-miR396c | chr5 | 831397185 | 831397342 | + | 831397205 | 831397225 | 831397302 | 831397322 | NO | 1180 |
| tch-miR166f | chr5 | 867247360 | 867247476 | + | 867247436 | 867247456 | 867247380 | 867247400 | YES | 12190 |
| tch-miR156d.1 | chr5 | 748178508 | 748178606 | + | 748178566 | 748178586 | 748178528 | 748178548 | YES | 390 |
| tch-miR156e.1 | chr5 | 748492800 | 748492898 | + | 748492858 | 748492878 | 748492820 | 748492840 | YES | 320 |
| tch-miR156f.1 | chr5 | 748750849 | 748750947 | + | 748750907 | 748750927 | 748750869 | 748750889 | YES | 360 |
| tch-miRN106a | chr5 | 19687064 | 19687169 | + | 19687129 | 19687149 | 19687084 | 19687104 | YES | 1210 |
| tch-miRN107 | chr5 | 71779171 | 71779295 | + | 71779255 | 71779275 | 71779191 | 71779211 | YES | 2570 |
| tch-miRN108 | chr5 | 101003308 | 101003425 | + | 101003385 | 101003405 | 101003328 | 101003348 | YES | 1649830 |
| tch-miRN109 | chr5 | 101666165 | 101666278 | + | 101666238 | 101666258 | 101666185 | 101666205 | YES | 667260 |
| tch-miRN110 | chr5 | 102200327 | 102200454 | - | 102200414 | 102200434 | 102200347 | 102200367 | YES | 1120 |
| tch-miRN111 | chr5 | 104863545 | 104863664 | - | 104863624 | 104863644 | 104863565 | 104863585 | YES | 90 |
| tch-miRN112a | chr5 | 152379473 | 152379593 | + | 152379493 | 152379513 | 152379553 | 152379573 | YES | 150 |
| tch-miRN113 | chr5 | 182277813 | 182277934 | + | 182277833 | 182277853 | 182277894 | 182277914 | YES | 100 |
| tch-miRN114 | chr5 | 229719336 | 229719491 | + | 229719356 | 229719376 | 229719451 | 229719471 | YES | 9310 |
| tch-miRN115 | chr5 | 231841885 | 231842000 | - | 231841905 | 231841925 | 231841960 | 231841980 | YES | 790 |
| tch-miRN40m | chr5 | 268324179 | 268324299 | + | 268324259 | 268324279 | 268324199 | 268324219 | YES | 190 |
| tch-miRN116 | chr5 | 301224069 | 301224221 | - | 301224089 | 301224109 | 301224181 | 301224201 | YES | 490 |
| tch-miRN117 | chr5 | 314639510 | 314639687 | - | 314639530 | 314639550 | 314639647 | 314639667 | YES | 640 |
| tch-miRN118 | chr5 | 315143251 | 315143370 | - | 315143271 | 315143291 | 315143330 | 315143350 | YES | 50 |
| tch-miRN40j | chr5 | 599851633 | 599851740 | - | 599851653 | 599851673 | 599851700 | 599851720 | YES | 180 |
| tch-miRN119 | chr5 | 720306782 | 720306906 | - | 720306802 | 720306822 | 720306866 | 720306886 | YES | 170 |
| tch-miRN120 | chr5 | 720535036 | 720535154 | - | 720535056 | 720535076 | 720535114 | 720535134 | YES | 80 |
| tch-miRN121a | chr5 | 759332248 | 759332363 | - | 759332323 | 759332343 | 759332268 | 759332288 | YES | 80 |
| tch-miRN122 | chr5 | 774451070 | 774451188 | - | 774451148 | 774451168 | 774451090 | 774451110 | YES | 8430 |
| tch-miRN123a | chr5 | 804327918 | 804328045 | + | 804328004 | 804328025 | 804327938 | 804327958 | YES | 86250 |
| tch-miRN123b | chr5 | 804409030 | 804409157 | + | 804409116 | 804409137 | 804409050 | 804409070 | YES | 118310 |
| tch-miRN124 | chr5 | 806448022 | 806448133 | - | 806448093 | 806448113 | 806448042 | 806448062 | YES | 1920 |
| tch-miR479a | chr6 | 10031466 | 10031578 | + | 10031486 | 10031507 | 10031537 | 10031558 | YES | 15150 |
| tch-miR171a | chr6 | 10381561 | 10381678 | - | 10381637 | 10381658 | 10381581 | 10381602 | YES | 510 |
| tch-miR156a | chr6 | 275038261 | 275038381 | + | 275038281 | 275038300 | 275038341 | 275038361 | YES | 356880 |
| tch-miR11892 | chr6 | 281020840 | 281020930 | - | 281020891 | 281020910 | 281020860 | 281020882 | NO | 20 |
| tch-miR166g | chr6 | 320694007 | 320694124 | + | 320694084 | 320694104 | 320694027 | 320694047 | YES | 50800 |
| tch-miR171b | chr6 | 527824302 | 527824425 | - | 527824322 | 527824343 | 527824384 | 527824405 | NO | 70 |
| tch-miR171c | chr6 | 584517294 | 584517415 | + | 584517375 | 584517395 | 584517314 | 584517334 | YES | 270 |
| tch-miR1874 | chr6 | 661962205 | 661962285 | + | 661962246 | 661962265 | 661962225 | 661962245 | NO | 30 |
| tch-miR1425 | chr6 | 837344692 | 837344848 | + | 837344712 | 837344732 | 837344808 | 837344828 | NO | 660 |
| tch-miR396d | chr6 | 869687139 | 869687283 | - | 869687243 | 869687263 | 869687159 | 869687179 | YES | 378950 |
| tch-miR479b | chr6 | 9188040 | 9188149 | + | 9188060 | 9188081 | 9188108 | 9188129 | YES | 80200 |
| tch-miR5078.1 | chr6 | 255797626 | 255797752 | - | 255797646 | 255797666 | 255797712 | 255797732 | YES | 2570 |
| tch-miR835.1 | chr6 | 255913135 | 255913275 | - | 255913155 | 255913175 | 255913233 | 255913255 | YES | 220 |
| tch-miR156g.1 | chr6 | 531985828 | 531985955 | + | 531985848 | 531985868 | 531985915 | 531985935 | YES | 1150 |
| tch-miR169o.1 | chr6 | 636338694 | 636338815 | - | 636338775 | 636338795 | 636338714 | 636338734 | YES | 80 |
| tch-miRN125 | chr6 | 3361599 | 3361742 | - | 3361619 | 3361639 | 3361702 | 3361722 | YES | 180 |
| tch-miRN126 | chr6 | 52417701 | 52417864 | + | 52417824 | 52417844 | 52417721 | 52417741 | YES | 860 |
| tch-miRN127 | chr6 | 63818818 | 63818916 | + | 63818876 | 63818896 | 63818838 | 63818858 | YES | 20900 |
| tch-miRN128 | chr6 | 111292541 | 111292643 | - | 111292561 | 111292581 | 111292603 | 111292623 | YES | 7660 |
| tch-miRN129 | chr6 | 300548173 | 300548366 | - | 300548326 | 300548346 | 300548193 | 300548213 | YES | 62680 |
| tch-miRN130 | chr6 | 305881861 | 305881964 | + | 305881924 | 305881944 | 305881881 | 305881901 | YES | 1490 |
| tch-miRN131 | chr6 | 360964788 | 360964968 | + | 360964808 | 360964828 | 360964928 | 360964948 | YES | 50 |
| tch-miRN132 | chr6 | 510220043 | 510220184 | + | 510220143 | 510220164 | 510220063 | 510220084 | YES | 9520 |
| tch-miRN133 | chr6 | 535611008 | 535611143 | + | 535611106 | 535611126 | 535611028 | 535611048 | YES | 1590 |
| tch-miRN134 | chr6 | 585377539 | 585377658 | + | 585377559 | 585377579 | 585377618 | 585377638 | YES | 400 |
| tch-miRN121b | chr6 | 599830733 | 599830847 | - | 599830807 | 599830827 | 599830753 | 599830773 | YES | 70 |
| tch-miRN135 | chr6 | 616669455 | 616669580 | + | 616669475 | 616669495 | 616669540 | 616669560 | YES | 130 |
| tch-miRN136 | chr6 | 634382094 | 634382207 | - | 634382167 | 634382187 | 634382114 | 634382135 | YES | 830 |
| tch-miRN137 | chr6 | 694855877 | 694856012 | + | 694855971 | 694855992 | 694855897 | 694855918 | YES | 340 |
| tch-miRN40g | chr6 | 728673324 | 728673422 | - | 728673386 | 728673406 | 728673344 | 728673364 | YES | 240 |
| tch-miRN138 | chr6 | 791376925 | 791377040 | - | 791376945 | 791376965 | 791377000 | 791377020 | YES | 350 |
| tch-miRN139 | chr6 | 866508393 | 866508610 | - | 866508570 | 866508590 | 866508413 | 866508433 | YES | 822160 |
| tch-miR8175b | chr7 | 189987584 | 189987707 | - | 189987604 | 189987624 | 189987666 | 189987687 | NO | 290 |
| tch-miR7814 | chr7 | 198856845 | 198857056 | + | 198856865 | 198856884 | 198857014 | 198857036 | NO | 20 |
| tch-miR482m | chr7 | 206143605 | 206143722 | + | 206143681 | 206143702 | 206143625 | 206143646 | YES | 101460 |
| tch-miR390c | chr7 | 224670964 | 224671071 | - | 224671031 | 224671051 | 224670984 | 224671004 | NO | 730 |
| tch-miR535b | chr7 | 45934201 | 45934321 | + | 45934221 | 45934241 | 45934281 | 45934301 | YES | 2923460 |
| tch-miR472b | chr7 | 745143064 | 745143167 | + | 745143126 | 745143147 | 745143084 | 745143104 | YES | 212630 |
| tch-miR169m | chr7 | 9735950 | 9736077 | + | 9736037 | 9736057 | 9735970 | 9735990 | YES | 26290 |
| tch-miR857.1 | chr7 | 650998569 | 650998688 | + | 650998589 | 650998609 | 650998648 | 650998668 | YES | 1760 |
| tch-miR157.1 | chr7 | 727310683 | 727310831 | + | 727310791 | 727310811 | 727310703 | 727310723 | YES | 90 |
| tch-miRN140 | chr7 | 13695173 | 13695300 | + | 13695193 | 13695213 | 13695260 | 13695280 | YES | 250 |
| tch-miRN40i | chr7 | 14124585 | 14124704 | - | 14124605 | 14124625 | 14124664 | 14124684 | YES | 310 |
| tch-miRN141 | chr7 | 14547169 | 14547299 | + | 14547259 | 14547279 | 14547189 | 14547209 | YES | 50 |
| tch-miRN40n | chr7 | 58588277 | 58588397 | + | 58588358 | 58588378 | 58588297 | 58588317 | YES | 680 |
| tch-miRN142 | chr7 | 96763204 | 96763317 | + | 96763277 | 96763297 | 96763224 | 96763244 | YES | 15160 |
| tch-miRN143 | chr7 | 121252048 | 121252203 | + | 121252068 | 121252088 | 121252163 | 121252183 | YES | 4060 |
| tch-miRN144 | chr7 | 145341433 | 145341550 | - | 145341510 | 145341530 | 145341453 | 145341473 | YES | 1840 |
| tch-miRN145 | chr7 | 194679662 | 194679794 | + | 194679682 | 194679703 | 194679754 | 194679774 | YES | 280760 |
| tch-miRN146 | chr7 | 194679891 | 194680008 | + | 194679967 | 194679988 | 194679911 | 194679932 | YES | 505590 |
| tch-miRN19b | chr7 | 225304616 | 225304758 | - | 225304718 | 225304738 | 225304636 | 225304656 | YES | 700 |
| tch-miRN147 | chr7 | 266559881 | 266560040 | - | 266560000 | 266560020 | 266559901 | 266559921 | YES | 36720 |
| tch-miRN148 | chr7 | 311080591 | 311080716 | + | 311080611 | 311080631 | 311080676 | 311080696 | YES | 260 |
| tch-miRN149 | chr7 | 350199740 | 350199863 | + | 350199823 | 350199843 | 350199760 | 350199780 | YES | 250 |
| tch-miRN150 | chr7 | 389694538 | 389694684 | - | 389694644 | 389694664 | 389694558 | 389694578 | YES | 1760 |
| tch-miRN151 | chr7 | 399573959 | 399574079 | - | 399574039 | 399574059 | 399573979 | 399573999 | YES | 230 |
| tch-miRN101b | chr7 | 491296275 | 491296429 | - | 491296295 | 491296316 | 491296389 | 491296409 | YES | 340 |
| tch-miRN152 | chr7 | 509722753 | 509722862 | - | 509722773 | 509722793 | 509722822 | 509722842 | YES | 280 |
| tch-miRN153 | chr7 | 571519843 | 571519941 | - | 571519863 | 571519883 | 571519901 | 571519921 | YES | 40 |
| tch-miRN154 | chr7 | 658332157 | 658332281 | + | 658332177 | 658332197 | 658332241 | 658332261 | YES | 240 |
| tch-miRN40h | chr7 | 700711265 | 700711383 | - | 700711343 | 700711363 | 700711285 | 700711304 | YES | 230 |
| tch-miRN155 | chr7 | 704476576 | 704476667 | + | 704476596 | 704476616 | 704476627 | 704476647 | YES | 80 |
| tch-miR477b | chr8 | 101021270 | 101021375 | - | 101021336 | 101021355 | 101021290 | 101021309 | NO | 20 |
| tch-miR477c | chr8 | 103776913 | 103777039 | - | 103777000 | 103777019 | 103776933 | 103776954 | NO | 290 |
| tch-miR171d | chr8 | 104750839 | 104750960 | - | 104750920 | 104750940 | 104750859 | 104750879 | YES | 510 |
| tch-miR390d | chr8 | 158170379 | 158170486 | + | 158170399 | 158170419 | 158170446 | 158170466 | YES | 820 |
| tch-miR529f | chr8 | 165970303 | 165970423 | + | 165970383 | 165970403 | 165970323 | 165970343 | YES | 900 |
| tch-miR399c | chr8 | 193630737 | 193630848 | - | 193630757 | 193630777 | 193630807 | 193630828 | YES | 420 |
| tch-miR399d | chr8 | 193917305 | 193917433 | - | 193917325 | 193917345 | 193917392 | 193917413 | NO | 70 |
| tch-miR399e | chr8 | 194096399 | 194096510 | - | 194096419 | 194096439 | 194096469 | 194096490 | YES | 340 |
| tch-miR399f | chr8 | 195170227 | 195170344 | + | 195170304 | 195170324 | 195170247 | 195170269 | NO | 110 |
| tch-miR894c | chr8 | 203106012 | 203106142 | - | 203106032 | 203106051 | 203106105 | 203106122 | NO | 30 |
| tch-miR6281 | chr8 | 324083182 | 324083426 | - | 324083202 | 324083222 | 324083385 | 324083406 | NO | 30 |
| tch-miR2118c | chr8 | 485043227 | 485043338 | + | 485043297 | 485043318 | 485043247 | 485043267 | YES | 37440 |
| tch-miR408a | chr8 | 489503084 | 489503209 | + | 489503168 | 489503189 | 489503104 | 489503124 | YES | 356200 |
| tch-miR408b | chr8 | 492754273 | 492754377 | + | 492754293 | 492754313 | 492754336 | 492754357 | YES | 15030 |
| tch-miR319d | chr8 | 5430577 | 5430811 | + | 5430772 | 5430791 | 5430597 | 5430616 | NO | 3010 |
| tch-miR159c | chr8 | 6875451 | 6875649 | + | 6875610 | 6875629 | 6875471 | 6875490 | YES | 3040 |
| tch-miR477d | chr8 | 92547961 | 92548075 | - | 92548034 | 92548055 | 92547981 | 92548001 | YES | 1240 |
| tch-miR477e | chr8 | 94452214 | 94452350 | + | 94452234 | 94452254 | 94452311 | 94452330 | YES | 1010 |
| tch-miR477f | chr8 | 94614840 | 94614976 | + | 94614860 | 94614880 | 94614937 | 94614956 | YES | 800 |
| tch-miR477g | chr8 | 96659623 | 96659759 | + | 96659643 | 96659663 | 96659720 | 96659739 | YES | 940 |
| tch-miR477h | chr8 | 96762903 | 96763039 | - | 96762999 | 96763019 | 96762923 | 96762942 | YES | 910 |
| tch-miR477i | chr8 | 97106903 | 97107010 | - | 97106970 | 97106990 | 97106923 | 97106943 | YES | 310 |
| tch-miR477j | chr8 | 99345811 | 99345947 | + | 99345831 | 99345851 | 99345908 | 99345927 | YES | 730 |
| tch-miR477k | chr8 | 99527743 | 99527879 | + | 99527763 | 99527783 | 99527840 | 99527859 | YES | 890 |
| tch-miR5648.1 | chr8 | 24974469 | 24974589 | + | 24974553 | 24974573 | 24974489 | 24974509 | YES | 140 |
| tch-miR477l.1 | chr8 | 93919633 | 93919771 | + | 93919653 | 93919673 | 93919731 | 93919751 | YES | 40 |
| tch-miR8742c.1 | chr8 | 95737250 | 95737388 | + | 95737348 | 95737368 | 95737270 | 95737291 | YES | 730 |
| tch-miR477m.1 | chr8 | 96576987 | 96577124 | + | 96577007 | 96577028 | 96577084 | 96577104 | YES | 160 |
| tch-miR8742a.1 | chr8 | 100618915 | 100619053 | - | 100618935 | 100618955 | 100619012 | 100619033 | YES | 900 |
| tch-miR8742b.1 | chr8 | 100629551 | 100629689 | - | 100629571 | 100629591 | 100629648 | 100629669 | YES | 690 |
| tch-miR9897.1 | chr8 | 276455314 | 276455428 | - | 276455405 | 276455425 | 276455334 | 276455354 | YES | 54510 |
| tch-miR7724.1 | chr8 | 414386627 | 414386764 | + | 414386724 | 414386744 | 414386647 | 414386667 | YES | 1280 |
| tch-miR1217.1 | chr8 | 720750640 | 720750803 | + | 720750660 | 720750680 | 720750763 | 720750783 | YES | 20640 |
| tch-miR160c.1 | chr8 | 754631753 | 754631856 | - | 754631773 | 754631793 | 754631816 | 754631836 | YES | 80820 |
| tch-miRN156 | chr8 | 33941725 | 33941844 | + | 33941745 | 33941765 | 33941804 | 33941824 | YES | 350 |
| tch-miRN157 | chr8 | 92550686 | 92550812 | + | 92550772 | 92550792 | 92550706 | 92550725 | YES | 1280 |
| tch-miRN158 | chr8 | 93102161 | 93102275 | - | 93102181 | 93102201 | 93102235 | 93102255 | YES | 150 |
| tch-miRN159 | chr8 | 97143504 | 97143611 | - | 97143524 | 97143544 | 97143571 | 97143591 | YES | 450 |
| tch-miRN160 | chr8 | 125532865 | 125532996 | + | 125532956 | 125532976 | 125532885 | 125532905 | YES | 9280 |
| tch-miRN161 | chr8 | 228454480 | 228454650 | + | 228454610 | 228454630 | 228454500 | 228454520 | YES | 330 |
| tch-miRN162 | chr8 | 406373982 | 406374100 | - | 406374002 | 406374022 | 406374060 | 406374080 | YES | 60 |
| tch-miRN163 | chr8 | 439867516 | 439867635 | + | 439867595 | 439867615 | 439867536 | 439867556 | YES | 2620 |
| tch-miRN40k | chr8 | 445200444 | 445200563 | - | 445200464 | 445200484 | 445200523 | 445200543 | YES | 460 |
| tch-miRN164 | chr8 | 570730663 | 570730787 | - | 570730683 | 570730703 | 570730747 | 570730767 | YES | 180 |
| tch-miRN165 | chr8 | 610829003 | 610829123 | + | 610829023 | 610829043 | 610829083 | 610829103 | YES | 1210 |
| tch-miRN166 | chr8 | 712349789 | 712349910 | + | 712349870 | 712349890 | 712349809 | 712349829 | YES | 61420 |
| tch-miR894d | chr9 | 217254372 | 217254481 | - | 217254392 | 217254411 | 217254441 | 217254461 | NO | 20 |
| tch-miR167 | chr9 | 329079029 | 329079145 | - | 329079105 | 329079125 | 329079049 | 329079069 | NO | 11120 |
| tch-miR2950b | chr9 | 3636699 | 3636815 | - | 3636775 | 3636795 | 3636719 | 3636739 | YES | 90 |
| tch-miR168c | chr9 | 531455678 | 531455788 | + | 531455698 | 531455718 | 531455748 | 531455768 | YES | 691180 |
| tch-miR398 | chr9 | 551573971 | 551574130 | - | 551573991 | 551574011 | 551574089 | 551574110 | NO | 30 |
| tch-miR894e | chr9 | 567774241 | 567774327 | + | 567774261 | 567774280 | 567774288 | 567774307 | NO | 20 |
| tch-miR529g | chr9 | 693423282 | 693423385 | - | 693423345 | 693423365 | 693423302 | 693423322 | YES | 2660 |
| tch-miR529h | chr9 | 693679601 | 693679720 | + | 693679621 | 693679641 | 693679680 | 693679700 | YES | 4100 |
| tch-miR529i | chr9 | 693685548 | 693685670 | + | 693685568 | 693685588 | 693685630 | 693685650 | YES | 7220 |
| tch-miR529j | chr9 | 694918272 | 694918394 | - | 694918354 | 694918374 | 694918292 | 694918312 | YES | 7520 |
| tch-miR156b | chr9 | 717836009 | 717836256 | - | 717836029 | 717836049 | 717836216 | 717836236 | NO | 150 |
| tch-miR906b.1 | chr9 | 194115477 | 194115596 | - | 194115497 | 194115517 | 194115556 | 194115576 | YES | 740 |
| tch-miR5303.1 | chr9 | 391261418 | 391261534 | - | 391261494 | 391261514 | 391261438 | 391261458 | YES | 50 |
| tch-miRN167 | chr9 | 139346218 | 139346377 | - | 139346238 | 139346258 | 139346337 | 139346357 | YES | 560 |
| tch-miRN168 | chr9 | 285978489 | 285978601 | + | 285978509 | 285978529 | 285978561 | 285978581 | YES | 700 |
| tch-miRN169 | chr9 | 417166954 | 417167073 | + | 417166974 | 417166994 | 417167033 | 417167053 | YES | 1030 |
| tch-miRN170 | chr9 | 441575591 | 441575709 | + | 441575611 | 441575631 | 441575669 | 441575689 | YES | 1790 |
| tch-miRN171 | chr9 | 541956582 | 541956699 | - | 541956602 | 541956622 | 541956659 | 541956679 | YES | 110 |
| tch-miRN172 | chr9 | 564695294 | 564695413 | - | 564695373 | 564695393 | 564695314 | 564695334 | YES | 1550 |
| tch-miRN173 | chr9 | 607120590 | 607120732 | + | 607120610 | 607120631 | 607120692 | 607120712 | YES | 3920 |
| tch-miRN174 | chr9 | 707072246 | 707072363 | - | 707072323 | 707072343 | 707072266 | 707072286 | YES | 110 |
| tch-miRN175 | chr9 | 709611622 | 709611730 | - | 709611690 | 709611710 | 709611642 | 709611662 | YES | 50 |
| tch-miRN176a | chr9 | 715017790 | 715017930 | + | 715017810 | 715017830 | 715017890 | 715017910 | YES | 120 |
| tch-miR156c.1 | JAHRHJ010000129.1 | 9353 | 9451 | + | 9411 | 9431 | 9373 | 9393 | YES | 360 |
| tch-miR11136 | JAHRHJ010000191.1 | 77383 | 77471 | - | 77441 | 77461 | 77403 | 77425 | NO | 20 |
| tch-miR396e.1 | JAHRHJ010000839.1 | 37269 | 37380 | + | 37289 | 37309 | 37340 | 37360 | YES | 700 |
| tch-miRN217 | JAHRHJ010002344.1 | 600455 | 600571 | + | 600531 | 600551 | 600475 | 600495 | YES | 180 |
| tch-miRN218 | JAHRHJ010003145.1 | 206833 | 206954 | + | 206914 | 206934 | 206853 | 206873 | YES | 50 |
| tch-miRN106b | JAHRHJ010003586.1 | 82823 | 82928 | + | 82888 | 82908 | 82843 | 82863 | YES | 1390 |
| tch-miRN219 | JAHRHJ010003738.1 | 76948 | 77071 | - | 76968 | 76988 | 77030 | 77051 | YES | 100 |

# Table S5 Distribution of known and novel miRNAs length in *Taxus chinensis*.

| Family | 20nt | 21nt | 22nt | 23nt | 24nt | Total |
| --- | --- | --- | --- | --- | --- | --- |
| known family | 36 | 141 | 28 | 2 | 1 | 208 |
| novel family | 4 | 217 | 31 | - | - | 252 |

# Table S6 Distribution of 5’ nucleotides of known and novel miRNAs in *Taxus chinensis*.

| Family | A | G | C | U |
| --- | --- | --- | --- | --- |
| known family | 39 | 24 | 32 | 113 |
| novel family | 62 | 20 | 44 | 126 |

# Table S7 Identified phasiRNAs in *Taxus chinensis*.

| **Name** | **P-val** | **Chr** | **Start** | **End** | **Best k-val** | **Phasi ratio** | **Max Tag Ratio** | **TPTM** | **MAT Abun** | **MAT2 Abun** |
| --- | --- | --- | --- | --- | --- | --- | --- | --- | --- | --- |
| Phas-1 | 5.00E-07 | 10 | 108533794 | 108534023 | 17 | 0.99 | 0.7 | 72870 | 5132 | 961 |
| Phas-2 | 1.00E-07 | 10 | 111632831 | 111633081 | 14 | 0.98 | 0.46 | 97560 | 4439 | 3372 |
| Phas-3 | 1.00E-06 | 10 | 119698513 | 119698879 | 14 | 0.96 | 0.4 | 25250 | 1007 | 699 |
| Phas-4 | 1.00E-06 | 10 | 120228861 | 120229363 | 14 | 0.77 | 0.21 | 32630 | 687 | 552 |
| Phas-5 | 5.00E-07 | 10 | 122052750 | 122052979 | 12 | 0.94 | 0.58 | 17460 | 1007 | 310 |
| Phas-6 | 1.00E-07 | 10 | 145447736 | 145447881 | 9 | 0.99 | 0.86 | 6460 | 558 | 31 |
| Phas-7 | 1.00E-07 | 10 | 145538509 | 145538780 | 11 | 0.99 | 0.71 | 7880 | 558 | 116 |
| Phas-8 | 1.00E-07 | 10 | 145671801 | 145672177 | 13 | 0.99 | 0.86 | 35040 | 3006 | 233 |
| Phas-9 | 1.00E-07 | 10 | 145750929 | 145751305 | 16 | 0.99 | 0.74 | 40690 | 3006 | 558 |
| Phas-10 | 1.00E-07 | 10 | 146402341 | 146402738 | 13 | 0.94 | 0.82 | 41330 | 3396 | 124 |
| Phas-12 | 1.00E-07 | 10 | 246940105 | 246940481 | 18 | 1 | 0.75 | 116860 | 8725 | 1411 |
| Phas-17 | 1.00E-07 | 10 | 277828525 | 277829048 | 14 | 0.94 | 0.25 | 202990 | 5090 | 3991 |
| Phas-18 | 1.00E-06 | 10 | 285850507 | 285850736 | 8 | 0.77 | 0.43 | 11900 | 515 | 230 |
| Phas-21 | 1.00E-07 | 10 | 304384520 | 304384791 | 16 | 0.95 | 0.68 | 47100 | 3225 | 313 |
| Phas-23 | 1.00E-07 | 10 | 314991519 | 314991811 | 13 | 0.98 | 0.53 | 10570 | 562 | 138 |
| Phas-25 | 1.00E-07 | 10 | 315656164 | 315656435 | 19 | 0.96 | 0.82 | 55130 | 4540 | 223 |
| Phas-27 | 1.00E-07 | 10 | 330182138 | 330182556 | 18 | 0.98 | 0.22 | 30230 | 652 | 595 |
| Phas-28 | 1.00E-07 | 10 | 331230812 | 331231209 | 18 | 0.99 | 0.57 | 94990 | 5385 | 1071 |
| Phas-29 | 1.00E-06 | 10 | 331880927 | 331881114 | 9 | 0.95 | 0.73 | 61270 | 4448 | 1071 |
| Phas-30 | 1.00E-07 | 10 | 343603425 | 343603612 | 11 | 0.86 | 0.32 | 11120 | 358 | 305 |
| Phas-31 | 1.00E-07 | 10 | 345575003 | 345575274 | 12 | 0.93 | 0.58 | 6150 | 358 | 81 |
| Phas-32 | 1.00E-07 | 10 | 345682764 | 345683035 | 12 | 0.93 | 0.58 | 6150 | 358 | 81 |
| Phas-33 | 1.00E-07 | 10 | 345937056 | 345937285 | 11 | 0.95 | 0.68 | 5250 | 358 | 65 |
| Phas-34 | 5.00E-07 | 10 | 346332497 | 346332726 | 10 | 0.97 | 0.32 | 15750 | 498 | 358 |
| Phas-35 | 1.00E-07 | 10 | 349439776 | 349440068 | 14 | 0.96 | 0.2 | 28270 | 577 | 427 |
| Phas-36 | 1.00E-07 | 10 | 352409631 | 352410007 | 13 | 0.96 | 0.28 | 40460 | 1125 | 628 |
| Phas-39 | 1.00E-06 | 10 | 365640989 | 365641260 | 12 | 0.96 | 0.38 | 54260 | 2080 | 2004 |
| Phas-41 | 1.00E-07 | 10 | 406133594 | 406134075 | 17 | 0.91 | 0.73 | 175990 | 12862 | 1032 |
| Phas-42 | 1.00E-07 | 10 | 406176342 | 406176739 | 18 | 0.93 | 0.73 | 175030 | 12862 | 1032 |
| Phas-43 | 1.00E-07 | 10 | 422106861 | 422107321 | 16 | 0.9 | 0.54 | 546270 | 29373 | 20149 |
| Phas-44 | 1.00E-07 | 10 | 422201597 | 422201784 | 7 | 0.98 | 0.86 | 82300 | 7106 | 635 |
| Phas-46 | 1.00E-07 | 10 | 444812051 | 444812679 | 10 | 0.96 | 0.63 | 22750 | 1431 | 182 |
| Phas-47 | 5.00E-07 | 10 | 444812450 | 444812679 | 8 | 0.99 | 0.56 | 25730 | 1431 | 675 |
| Phas-51 | 1.00E-07 | 10 | 471011307 | 471011662 | 11 | 0.99 | 0.63 | 19700 | 1240 | 548 |
| Phas-52 | 5.00E-07 | 10 | 486547351 | 486547580 | 8 | 1 | 0.46 | 11000 | 501 | 278 |
| Phas-54 | 1.00E-07 | 10 | 486886192 | 486886505 | 12 | 0.96 | 0.32 | 15630 | 501 | 432 |
| Phas-56 | 5.00E-07 | 10 | 495637828 | 495638057 | 9 | 1 | 0.61 | 11410 | 694 | 245 |
| Phas-57 | 1.00E-07 | 10 | 523853373 | 523853602 | 12 | 0.99 | 0.58 | 18500 | 1075 | 689 |
| Phas-58 | 1.00E-07 | 10 | 523985692 | 523986068 | 16 | 0.99 | 0.29 | 101600 | 2931 | 2626 |
| Phas-60 | 1.00E-07 | 10 | 535385815 | 535386002 | 16 | 0.96 | 0.37 | 243820 | 9027 | 4412 |
| Phas-61 | 1.00E-07 | 10 | 535433839 | 535434068 | 22 | 0.98 | 0.78 | 340370 | 26558 | 3640 |
| Phas-62 | 1.00E-07 | 10 | 535434539 | 535434747 | 15 | 0.98 | 0.37 | 216500 | 8009 | 4021 |
| Phas-63 | 5.00E-07 | 10 | 535714609 | 535714838 | 21 | 0.98 | 0.68 | 387990 | 26558 | 3649 |
| Phas-64 | 1.00E-07 | 10 | 535715202 | 535715410 | 17 | 0.85 | 0.59 | 278920 | 16425 | 5162 |
| Phas-66 | 1.00E-07 | 10 | 542385984 | 542386213 | 11 | 0.9 | 0.64 | 37310 | 2406 | 620 |
| Phas-67 | 1.00E-07 | 10 | 544523825 | 544524138 | 21 | 0.99 | 0.33 | 934420 | 31001 | 18770 |
| Phas-68 | 1.00E-07 | 10 | 544656852 | 544657165 | 20 | 0.98 | 0.4 | 471870 | 18770 | 11615 |
| Phas-70 | 1.00E-07 | 10 | 586997193 | 586997548 | 16 | 0.99 | 0.3 | 22390 | 673 | 416 |
| Phas-90 | 1.00E-07 | 11 | 101386742 | 101387097 | 13 | 0.91 | 0.25 | 25750 | 649 | 520 |
| Phas-91 | 1.00E-07 | 11 | 117492376 | 117492752 | 17 | 0.91 | 0.42 | 47720 | 1996 | 831 |
| Phas-92 | 5.00E-07 | 11 | 138800830 | 138801059 | 14 | 0.99 | 0.43 | 391920 | 16906 | 8313 |
| Phas-94 | 1.00E-07 | 11 | 171316569 | 171316945 | 14 | 0.98 | 0.71 | 304060 | 21542 | 5011 |
| Phas-95 | 1.00E-07 | 11 | 195846289 | 195846476 | 9 | 0.95 | 0.46 | 342800 | 15783 | 14994 |
| Phas-96 | 5.00E-07 | 11 | 360979102 | 360979310 | 8 | 1 | 0.27 | 23390 | 621 | 616 |
| Phas-98 | 5.00E-07 | 1 | 110160753 | 110161003 | 18 | 0.91 | 0.25 | 669150 | 16897 | 13633 |
| Phas-99 | 1.00E-07 | 1 | 110570910 | 110571118 | 14 | 0.75 | 0.29 | 109730 | 3202 | 2961 |
| Phas-100 | 5.00E-07 | 1 | 110804932 | 110805182 | 10 | 0.98 | 0.5 | 7950 | 401 | 157 |
| Phas-104 | 1.00E-06 | 1 | 289856397 | 289856731 | 15 | 0.95 | 0.47 | 58920 | 2760 | 1357 |
| Phas-106 | 1.00E-07 | 1 | 290258463 | 290258713 | 14 | 0.99 | 0.47 | 208060 | 9831 | 4234 |
| Phas-107 | 1.00E-07 | 1 | 295655245 | 295655672 | 19 | 0.98 | 0.51 | 168610 | 8588 | 989 |
| Phas-111 | 1.00E-07 | 11 | 534536393 | 534536622 | 9 | 0.99 | 0.51 | 6360 | 323 | 266 |
| Phas-113 | 1.00E-07 | 11 | 552652548 | 552652756 | 17 | 0.89 | 0.39 | 18370 | 715 | 235 |
| Phas-117 | 1.00E-07 | 11 | 585089509 | 585089759 | 16 | 0.99 | 0.3 | 17840 | 531 | 458 |
| Phas-119 | 1.00E-06 | 11 | 645036033 | 645036241 | 11 | 0.82 | 0.72 | 36030 | 2578 | 395 |
| Phas-120 | 5.00E-07 | 11 | 645109632 | 645109882 | 10 | 0.82 | 0.89 | 28930 | 2578 | 142 |
| Phas-121 | 1.00E-07 | 11 | 645687590 | 645687861 | 12 | 0.92 | 0.76 | 63770 | 4829 | 307 |
| Phas-124 | 1.00E-07 | 11 | 646127050 | 646127321 | 13 | 0.93 | 0.58 | 194650 | 11226 | 3181 |
| Phas-125 | 1.00E-07 | 11 | 646136755 | 646136984 | 11 | 0.96 | 0.34 | 14280 | 490 | 365 |
| Phas-126 | 5.00E-07 | 11 | 646304750 | 646304979 | 19 | 0.89 | 0.42 | 137370 | 5737 | 2789 |
| Phas-127 | 1.00E-07 | 12 | 9855204 | 9855454 | 15 | 0.95 | 0.42 | 28850 | 1222 | 491 |
| Phas-128 | 1.00E-07 | 12 | 16500742 | 16501013 | 14 | 0.99 | 0.51 | 63650 | 3274 | 1304 |
| Phas-131 | 5.00E-07 | 12 | 65562592 | 65562821 | 7 | 1 | 0.88 | 38520 | 3396 | 124 |
| Phas-132 | 1.00E-07 | 12 | 65706439 | 65707164 | 19 | 0.88 | 0.5 | 67410 | 3396 | 801 |
| Phas-133 | 1.00E-07 | 12 | 65713048 | 65713382 | 19 | 0.93 | 0.63 | 136000 | 8588 | 989 |
| Phas-135 | 5.00E-07 | 12 | 65954806 | 65955014 | 10 | 0.95 | 0.88 | 38560 | 3396 | 124 |
| Phas-136 | 1.00E-07 | 12 | 65960852 | 65961102 | 10 | 0.86 | 0.31 | 32350 | 989 | 916 |
| Phas-139 | 1.00E-07 | 12 | 237289270 | 237289667 | 13 | 0.99 | 0.8 | 86420 | 6944 | 1142 |
| Phas-140 | 1.00E-07 | 12 | 326696348 | 326696619 | 10 | 0.96 | 0.51 | 13040 | 659 | 190 |
| Phas-141 | 1.00E-07 | Ctg9685 | 9668770 | 9669041 | 16 | 0.95 | 0.68 | 47100 | 3225 | 313 |
| Phas-145 | 1.00E-07 | Ctg9685 | 74602356 | 74602690 | 15 | 0.99 | 0.41 | 242420 | 9831 | 4234 |
| Phas-147 | 5.00E-07 | Ctg9685 | 169020593 | 169020822 | 9 | 0.84 | 0.57 | 13190 | 754 | 208 |
| Phas-148 | 1.00E-07 | Ctg9685 | 203925602 | 203925894 | 12 | 0.99 | 0.36 | 21180 | 754 | 404 |
| Phas-150 | 1.00E-07 | Ctg9685 | 306832182 | 306832684 | 21 | 0.97 | 0.48 | 381970 | 18458 | 6388 |
| Phas-153 | 1.00E-07 | Ctg9685 | 383854134 | 383854489 | 16 | 0.99 | 0.29 | 23390 | 673 | 416 |
| Phas-155 | 1.00E-07 | 1 | 594954220 | 594954617 | 21 | 0.84 | 0.19 | 206890 | 3994 | 2338 |
| Phas-156 | 1.00E-07 | 1 | 599156002 | 599156252 | 15 | 0.91 | 0.35 | 19640 | 686 | 598 |
| Phas-158 | 1.00E-07 | 1 | 666606829 | 666607142 | 16 | 0.87 | 0.38 | 451180 | 16968 | 14822 |
| Phas-159 | 1.00E-07 | 1 | 707463056 | 707463201 | 8 | 0.95 | 0.74 | 4480 | 330 | 81 |
| Phas-160 | 5.00E-07 | 1 | 714313341 | 714313570 | 12 | 1 | 0.46 | 12860 | 591 | 274 |
| Phas-162 | 1.00E-06 | 1 | 732853161 | 732853579 | 21 | 0.89 | 0.49 | 1170430 | 57530 | 22522 |
| Phas-163 | 1.00E-07 | 1 | 733000321 | 733000676 | 21 | 0.76 | 0.26 | 2895650 | 75868 | 57530 |
| Phas-164 | 1.00E-07 | 1 | 733134982 | 733135253 | 18 | 0.92 | 0.66 | 1148610 | 75868 | 27191 |
| Phas-165 | 1.00E-07 | 1 | 733303852 | 733304081 | 16 | 0.8 | 0.45 | 1489270 | 66572 | 58886 |
| Phas-167 | 5.00E-07 | 1 | 784570072 | 784570301 | 13 | 0.95 | 0.63 | 15680 | 991 | 206 |
| Phas-169 | 1.00E-07 | 2 | 30569415 | 30569686 | 11 | 0.99 | 0.32 | 10950 | 350 | 206 |
| Phas-174 | 1.00E-06 | 2 | 751501613 | 751501842 | 13 | 0.95 | 0.49 | 48860 | 2382 | 1992 |
| Phas-175 | 1.00E-07 | 2 | 752151177 | 752151427 | 14 | 0.9 | 0.4 | 59980 | 2382 | 2350 |
| Phas-177 | 5.00E-07 | 2 | 752621700 | 752621929 | 11 | 0.88 | 0.4 | 58910 | 2382 | 2350 |
| Phas-178 | 1.00E-07 | 2 | 850239972 | 850240201 | 21 | 0.98 | 0.82 | 139530 | 11504 | 1265 |
| Phas-182 | 1.00E-06 | 3 | 107453661 | 107453911 | 9 | 0.95 | 0.51 | 24510 | 1244 | 213 |
| Phas-184 | 1.00E-07 | 3 | 125018611 | 125018924 | 9 | 0.91 | 0.62 | 8310 | 518 | 145 |
| Phas-185 | 1.00E-07 | 3 | 125810601 | 125810893 | 9 | 0.91 | 0.65 | 7920 | 518 | 145 |
| Phas-187 | 1.00E-07 | 3 | 137833065 | 137833294 | 15 | 0.91 | 0.29 | 99810 | 2934 | 2932 |
| Phas-188 | 1.00E-07 | 3 | 137833609 | 137833754 | 10 | 0.87 | 0.64 | 181500 | 11696 | 5148 |
| Phas-189 | 1.00E-07 | 3 | 153151108 | 153151421 | 15 | 1 | 0.63 | 43120 | 2714 | 783 |
| Phas-190 | 1.00E-07 | 3 | 196780812 | 196781125 | 15 | 0.93 | 0.54 | 14050 | 762 | 189 |
| Phas-191 | 1.00E-07 | 3 | 197611197 | 197611510 | 15 | 0.94 | 0.42 | 18060 | 762 | 584 |
| Phas-194 | 1.00E-07 | 3 | 718216073 | 718216302 | 9 | 0.91 | 0.8 | 30010 | 2406 | 429 |
| Phas-195 | 1.00E-07 | 3 | 718285362 | 718285696 | 12 | 0.92 | 0.68 | 35430 | 2406 | 429 |
| Phas-196 | 1.00E-07 | 3 | 718310865 | 718311199 | 12 | 0.91 | 0.58 | 41210 | 2406 | 620 |
| Phas-198 | 5.00E-07 | 3 | 849184034 | 849184200 | 10 | 0.98 | 0.43 | 71040 | 3080 | 2426 |
| Phas-205 | 1.00E-07 | 4 | 593810752 | 593810960 | 9 | 0.98 | 0.34 | 11110 | 380 | 363 |
| Phas-206 | 5.00E-07 | 4 | 653084238 | 653084467 | 12 | 0.99 | 0.63 | 33340 | 2094 | 853 |
| Phas-209 | 5.00E-07 | 4 | 769793093 | 769793385 | 14 | 0.81 | 0.66 | 1362750 | 89953 | 22406 |
| Phas-211 | 5.00E-07 | 4 | 839963844 | 839964031 | 10 | 0.97 | 0.75 | 6330 | 473 | 63 |
| Phas-214 | 1.00E-07 | 4 | 874297754 | 874298506 | 11 | 0.86 | 0.56 | 60600 | 3396 | 801 |
| Phas-215 | 1.00E-07 | 5 | 7808409 | 7808764 | 9 | 0.97 | 0.43 | 45750 | 1979 | 1040 |
| Phas-216 | 1.00E-07 | 5 | 8397245 | 8397474 | 18 | 0.89 | 0.38 | 18800 | 715 | 235 |
| Phas-217 | 1.00E-07 | 5 | 8638491 | 8638678 | 13 | 0.77 | 0.58 | 275530 | 16094 | 4631 |
| Phas-218 | 1.00E-07 | 5 | 42933049 | 42933404 | 18 | 0.78 | 0.8 | 3716640 | 296339 | 39279 |
| Phas-219 | 1.00E-07 | 5 | 42941573 | 42941928 | 19 | 0.91 | 0.47 | 396190 | 18466 | 11302 |
| Phas-220 | 1.00E-07 | 5 | 42952152 | 42952507 | 21 | 0.98 | 0.88 | 1742540 | 153792 | 4461 |
| Phas-221 | 1.00E-07 | 5 | 43007464 | 43007819 | 19 | 0.96 | 0.27 | 417100 | 11398 | 5449 |
| Phas-222 | 1.00E-07 | 5 | 43072581 | 43073020 | 20 | 0.87 | 0.55 | 1227460 | 67463 | 11726 |
| Phas-224 | 1.00E-07 | 5 | 43275761 | 43276263 | 21 | 0.97 | 0.48 | 381970 | 18458 | 6388 |
| Phas-225 | 1.00E-07 | 5 | 43399602 | 43399978 | 18 | 0.88 | 0.37 | 470940 | 17510 | 14568 |
| Phas-227 | 1.00E-07 | 5 | 104436118 | 104436410 | 21 | 0.98 | 0.48 | 59620 | 2858 | 1328 |
| Phas-228 | 1.00E-07 | 5 | 104855427 | 104855761 | 13 | 0.91 | 0.44 | 135380 | 6008 | 4114 |
| Phas-237 | 1.00E-07 | 6 | 104713182 | 104713411 | 8 | 0.99 | 0.7 | 122050 | 8602 | 1258 |
| Phas-238 | 1.00E-07 | 6 | 104903234 | 104903526 | 12 | 0.9 | 0.42 | 221700 | 9411 | 8602 |
| Phas-239 | 5.00E-07 | 6 | 105355698 | 105355927 | 11 | 0.98 | 0.5 | 17200 | 856 | 584 |
| Phas-240 | 1.00E-07 | 6 | 106098100 | 106098308 | 10 | 1 | 0.34 | 25550 | 856 | 584 |
| Phas-241 | 1.00E-07 | 6 | 107867298 | 107867527 | 8 | 0.99 | 0.7 | 122050 | 8602 | 1258 |
| Phas-242 | 1.00E-07 | 6 | 108062516 | 108062892 | 9 | 0.96 | 0.74 | 116870 | 8602 | 1258 |
| Phas-243 | 1.00E-07 | 6 | 109576574 | 109576845 | 19 | 0.99 | 0.89 | 83350 | 7424 | 393 |
| Phas-245 | 1.00E-07 | 6 | 596758260 | 596758552 | 17 | 0.99 | 0.88 | 542120 | 47948 | 1227 |
| Phas-246 | 1.00E-07 | 6 | 596758887 | 596759074 | 11 | 0.98 | 0.44 | 268740 | 11768 | 8094 |
| Phas-249 | 1.00E-07 | 6 | 683682908 | 683683200 | 13 | 0.98 | 0.43 | 17440 | 754 | 393 |
| Phas-253 | 1.00E-07 | 6 | 752087099 | 752087307 | 16 | 0.95 | 0.48 | 693040 | 33123 | 15935 |
| Phas-255 | 5.00E-07 | 6 | 752361472 | 752361764 | 14 | 0.9 | 0.35 | 24520 | 855 | 581 |
| Phas-256 | 1.00E-07 | 6 | 752662281 | 752662510 | 10 | 0.96 | 0.54 | 6870 | 371 | 135 |
| Phas-257 | 1.00E-07 | 6 | 752953609 | 752953775 | 12 | 0.92 | 0.64 | 7200 | 462 | 111 |
| Phas-262 | 5.00E-07 | 6 | 862533410 | 862533744 | 10 | 0.83 | 0.5 | 39340 | 1952 | 507 |
| Phas-263 | 1.00E-07 | 6 | 863571932 | 863572161 | 11 | 0.95 | 0.7 | 27950 | 1952 | 507 |
| Phas-264 | 1.00E-07 | 6 | 889198808 | 889199205 | 12 | 0.94 | 0.52 | 67860 | 3506 | 1139 |
| Phas-265 | 1.00E-07 | 7 | 2062919 | 2063274 | 20 | 0.96 | 0.41 | 423230 | 17421 | 6448 |
| Phas-268 | 1.00E-07 | 7 | 597583981 | 597584126 | 9 | 0.87 | 0.54 | 14690 | 800 | 195 |
| Phas-270 | 1.00E-07 | 7 | 721553535 | 721553932 | 18 | 0.96 | 0.32 | 19710 | 636 | 280 |
| Phas-272 | 1.00E-07 | 7 | 775487947 | 775488365 | 15 | 0.98 | 0.29 | 14000 | 402 | 269 |
| Phas-274 | 1.00E-07 | 7 | 782344304 | 782344575 | 13 | 0.97 | 0.24 | 110470 | 2692 | 2655 |
| Phas-275 | 1.00E-06 | 8 | 182544090 | 182544298 | 7 | 0.9 | 0.71 | 6180 | 439 | 58 |
| Phas-276 | 1.00E-07 | 8 | 372882344 | 372882678 | 19 | 0.98 | 0.54 | 40670 | 2198 | 628 |
| Phas-277 | 5.00E-07 | 8 | 647108274 | 647108608 | 9 | 0.97 | 0.43 | 220240 | 9411 | 8602 |
| Phas-278 | 1.00E-07 | 8 | 694094764 | 694095014 | 13 | 0.99 | 0.58 | 9430 | 544 | 177 |
| Phas-284 | 1.00E-07 | 8 | 705646094 | 705646323 | 7 | 1 | 0.64 | 7840 | 498 | 132 |
| Phas-286 | 1.00E-07 | 9 | 558021766 | 558021995 | 12 | 0.99 | 0.4 | 11490 | 456 | 214 |
| Phas-287 | 1.00E-07 | 9 | 606166718 | 606167052 | 7 | 0.93 | 0.65 | 5100 | 330 | 74 |
| Phas-288 | 1.00E-07 | 9 | 606342827 | 606343140 | 10 | 0.96 | 0.6 | 5540 | 330 | 81 |
| Phas-290 | 1.00E-07 | 9 | 662647339 | 662647862 | 14 | 0.99 | 0.48 | 50960 | 2457 | 416 |
| Phas-297 | 1.00E-07 | 9 | 754904793 | 754905064 | 16 | 0.99 | 0.21 | 50710 | 1084 | 1046 |

# Table S8 Target genes of miRNAs.

| miRNA ID | target ID |  | miRNA ID | target ID |
| --- | --- | --- | --- | --- |
| miR1083 | GGPPS-2 |  | miRN104 | BAPT-1 |
| miR11309 | T10βOH_like_12 |  | miRN104 | TBT-2 |
| miR11485 | T7βOH-1 |  | miRN108 | TBT-2 |
| miR11534 | T10βOH_like_1 |  | miRN108 | TXS-2 |
| miR1425 | TXS-2 |  | miRN109 | TBT-2 |
| miR1536.1 | T13αOH_like_2 |  | miRN110 | GGPPS-1 |
| miR156c.1 | CoA Ligase-2 |  | miRN111 | CoA Ligase-4 |
| miR156c.1 | T13αOH-2 |  | miRN113 | BAPT-4 |
| miR156d.1 | CoA Ligase-2 |  | miRN114 | T10βOH_like_4 |
| miR156d.1 | T13αOH-2 |  | miRN118 | GGPPS-4 |
| miR156e.1 | CoA Ligase-2 |  | miRN134 | T5αOH_like_5 |
| miR156e.1 | T13αOH-2 |  | miRN137 | T5αOH-4 |
| miR156f.1 | CoA Ligase-2 |  | miRN143 | T10βOH_like_7 |
| miR156f.1 | T13αOH-2 |  | miRN143 | T10βOH_like_15 |
| miR159b | T5αOH_like_5 |  | miRN148 | TB506-2 |
| miR164a | T10βOH_like_8 |  | miRN151 | T10βOH_like_20 |
| miR164b | T10βOH_like_8 |  | miRN157 | TB506-1 |
| miR164c | T10βOH_like_8 |  | miRN169 | T5αOH_like_6 |
| miR166a | TB506-1 |  | miRN175 | T10βOH_like_1 |
| miR166c | TB506-1 |  | miRN176a | T5αOH-1 |
| miR166d | TB506-1 |  | miRN176a | T5αOH-3 |
| miR166e | TB506-1 |  | miRN185 | CoA Ligase-4 |
| miR166f | TB506-1 |  | miRN185 | T10βOH_like_13 |
| miR166g | TB506-1 |  | miRN192 | T5αOH-4 |
| miR169n.1 | T10βOH_like_2 |  | miRN196 | T10βOH_like_6 |
| miR169o.1 | TXS-1 |  | miRN196 | T10βOH_like_14 |
| miR1871 | CoA Ligase-4 |  | miRN196 | T5αOH_like_8 |
| miR1874 | BAPT-3 |  | miRN196 | T10βOH_like_17 |
| miR393 | T13αOH-2 |  | miRN21 | BAPT-1 |
| miR393 | T13αOH-3 |  | miRN21 | TBT-1 |
| miR393 | T5αOH-3 |  | miRN217 | PAM-1 |
| miR393 | TBT-3 |  | miRN219 | TB506-1 |
| miR394 | T5αOH_like_4 |  | miRN32 | T5αOH_like_10 |
| miR396b | GGPPS-4 |  | miRN36 | T5αOH_like_5 |
| miR396c | GGPPS-4 |  | miRN40i | T5αOH_like_12 |
| miR396d | GGPPS-4 |  | miRN40j | T5αOH_like_12 |
| miR397d | T10βOH_like_7 |  | miRN40k | T5αOH_like_12 |
| miR472a | T13αOH_like_2 |  | miRN40n | T10βOH-2 |
| miR479b | T5αOH_like_5 |  | miRN40n | TBT-1 |
| miR482a | GGPPS-4 |  | miRN5 | T7βOH-1 |
| miR482b | GGPPS-4 |  | miRN52a | CoA Ligase-4 |
| miR482f | TB506-1 |  | miRN52b | CoA Ligase-4 |
| miR482g | T5αOH_like_4 |  | miRN52c | CoA Ligase-4 |
| miR482j | TXS-2 |  | miRN54 | T10βOH_like_4 |
| miR535b | T10βOH_like_2 |  | miRN54 | T13αOH-1 |
| miR5780 | T5αOH_like_2 |  | miRN55 | T10βOH_like_8 |
| miR5780 | T5αOH_like_9 |  | miRN65 | T5αOH-3 |
| miR5780 | T5αOH-1 |  | miRN78 | T10βOH_like_8 |
| miR5780 | T5αOH-3 |  | miRN9 | T13αOH-1 |
| miR6273 | T13αOH-2 |  | miRN92 | GGPPS-1 |
| miR7762.1 | DBTNBT-1 |  | miRN97 | T10βOH_like_12 |
| miR7762.1 | DBTNBT-2 |  | miRN97 | T10βOH_like_18 |
| miR7762.1 | TAT-1 |  | miRN97 | T10βOH_like_20 |
| miR8742a.1 | PAM-1 |  | miRN99 | TXS-1 |
| miR8742b.1 | PAM-1 |  | miRN99 | TXS-2 |
| miR8742c.1 | PAM-1 |  |  |  |
| miR894a | TB506-2 |  |  |  |
| miR894c | TB506-2 |  |  |  |
| miR9722 | T13αOH_like_2 |  |  |  |

# Table S9 Target genes of phasiRNAs.

| phasiRNA ID | target ID |
| --- | --- |
| Phas-100 | T10βOH_like_9 |
| Phas-137 | T10βOH_like_14 |
| Phas-137 | T5αOH_like_7 |
| Phas-14 | T5αOH_like_4 |
| Phas-159 | T10βOH_like_5 |
| Phas-160 | T10βOH_like_9 |
| Phas-161 | GGPPS-1 |
| Phas-162 | T10βOH_like_9 |
| Phas-184 | T10βOH_like_8 |
| Phas-193 | CoA Ligase-1 |
| Phas-203 | T13αOH-2 |
| Phas-204 | T13αOH-2 |
| Phas-217 | T5αOH_like_4 |
| Phas-227 | T10βOH_like_1 |
| Phas-24 | T13αOH_like_2 |
| Phas-26 | T10βOH_like_1 |
| Phas-269 | T5αOH_like_7 |
| Phas-270 | T10βOH_like_1 |
| Phas-284 | CoA Ligase-2 |
| Phas-297 | T10βOH_like_1 |
| Phas-3 | T10βOH_like_1 |
| Phas-3 | T5αOH_like_10 |
| Phas-49 | TB506-1 |
| Phas-65 | T7βOH_like_1 |
| Phas-65 | T7βOH-1 |
| Phas-69 | DBTNBT-2 |
| Phas-82 | T5αOH_like_1 |
| Phas-98 | T10βOH_like_14 |
| Phas-98 | T5αOH_like_7 |
| Phas-99 | T10βOH_like_9 |
